# Inhibition of the NOTCH1 Pathway in the Stressed Heart Limits Fibrosis and Promotes Recruitment of Non-Myocyte Cells into the Cardiomyocyte Fate

**DOI:** 10.3390/jcdd9040111

**Published:** 2022-04-07

**Authors:** Mohamed Nemir, Maryam Kay, Damien Maison, Corinne Berthonneche, Alexandre Sarre, Isabelle Plaisance, Thierry Pedrazzini

**Affiliations:** 1 Experimental Cardiology Unit, Division of Cardiology, Department of Cardiovascular Medicine, University of Lausanne Medical School, 1011 Lausanne, Switzerland; mohamed.nemir@chuv.ch (M.N.); maryam.kay@chuv.ch (M.K.); damien.maison@chuv.ch (D.M.); isabelle.plaisance@chuv.ch (I.P.); 2 Cardiovascular Assessment Facility, University of Lausanne, 1011 Lausanne, Switzerland; corinne.berthonneche@chuv.ch (C.B.); alexandre.sarre@chuv.ch (A.S.)

**Keywords:** heart, remodeling, fibrosis, cardiomyocyte production, NOTCH

## Abstract

Cardiac pathologies lead to an acute or gradual loss of cardiomyocytes. Because of the limited regenerative capacity of the mammalian heart, cardiomyocytes are only replaced by fibrotic tissue. Excessive fibrosis contributes to the deterioration of cardiac function and the transition to heart failure, which is the leading cause of morbidity and mortality worldwide. Currently, no treatments can promote replenishment of the injured heart with newly formed cardiomyocytes. In this context, regenerative strategies explore the possibility to promote recovery through induction of cardiomyocyte production from pre-existing cardiomyocytes. On the other hand, cardiac non-myocyte cells can be directly reprogrammed into induced cardiac precursor cells and cardiomyocytes, suggesting that these cells could be exploited to produce cardiomyocytes in vivo. Here, we provide evidence that the sequential activation and inhibition of the NOTCH1 signaling pathway in the stressed heart decreases fibrosis and improves cardiac function in the stressed heart. This is accompanied by the emergence of new cardiomyocytes from non-myocyte origin. Overall, our data show how a developmental pathway such as the NOTCH pathway can be manipulated to provide therapeutic benefit in the damaged heart.

## 1. Introduction

In the adult heart, the rate of cardiomyocyte (CM) renewal is low [1,2,3]. In case of ischemic injury, which leads to massive CM death, the replacement of the lost myocardial tissue is insufficient to restore cardiac function. A default repair process leads to the formation of a fibrotic scar, stimulation of interstitial fibrosis and a gradual transition to heart failure [4]. Nevertheless, recent evidence suggests that the adult mammalian heart is capable of myocyte formation through division of preexisting CMs [1,2,3]. However, enhancing endogenous mechanisms of CM formation in the damaged or failing heart remains a major challenge [5,6]. Current efforts are focused on promoting CM proliferation, on stimulating cardiogenic differentiation in pluripotent and multipotent precursors [5,6,7,8], and on reprogramming of cardiac fibroblasts into CMs [9,10]. In this context, the contribution of resident cardiac precursor cells (CPCs) is highly debated [11].

Cardiac damage triggers rapid CM hypertrophy to cope with the increased hemodynamic workload. This occurs in case of pressure overload as observed in the transaortic constriction (TAC) model [12]. In addition, cardiac fibroblasts are activated in the injured heart, proliferate and differentiate into myofibroblasts [13]. The latter mediate deposition of extracellular matrix, which eventually leads to cardiac fibrosis, contributing to increased stiffness and dysfunction [14]. Myofibroblast differentiation is controlled by exogenous and cell-autonomous factors, including recently discovered long noncoding RNAs (lncRNAs). In particular, *Wisper* (*Wisp2* super-enhancer–associated RNA) is a lncRNA that controls the fibroblastic gene program in stimulated cardiac fibroblasts [15]. *Wisper* regulates myofibroblast differentiation, survival, proliferation and migration. In vivo, *Wisper* knockdown inhibits cardiac fibrosis and prevents the deterioration of cardiac function after myocardial infarction [15].

The NOTCH signaling pathway is also activated in the stressed heart. This occurs secondary to interaction of transmembrane ligands (JAGGED1, 2, and DELTA-LIKE1, 3 and 4) and receptors (NOTCH1–4). NOTCH receptor engagement leads to a conformational change of the negative regulatory region (NRR) of the protein [16]. This allows cleavage of its extracellular domain by an ADAM protease [17]. Subsequently, a second cleavage by a γ-secretase leads to the release of the intracellular domain of the receptor, which translocates to the nucleus, associates with the DNA-binding protein RBP-Jκ and activates target gene expression such as HES1, HEY1 and HEY2 [17]. Consistently, NRR blockade using therapeutic antibodies targeting NOTCH1 (anti-NNR1; αNRR1) or NOTCH2 (anti-NRR2; αNRR2) interferes with the ADAM protease-mediated S2 cleavage and inhibits NOTCH activation [18].

The NOTCH signaling pathway exerts stage-specific actions during heart development [19,20]. In CMs, NOTCH plays important roles during specification, and notably inhibits terminal differentiation [21,22,23,24,25]. NOTCH downregulation is therefore necessary for the production of functional CMs in the developing myocardium. In addition, NOTCH is a crucial regulator of adverse CM hypertrophy in the adult heart [26,27,28]. NOTCH1, NOTCH2 and JAGGED1 are the predominant members of the NOTCH family expressed in adulthood. We have previously shown that the hypertrophic and fibrotic response to pressure overload was reduced in transgenic mice overexpressing *Jagged1* in CMs [28]. This occurred concomitantly with an expansion of *Nkx2*–5^+^ CPCs. This situation was not associated with stimulated CM formation, likely because chronic NOTCH stimulation prevented further CPC differentiation into terminally differentiated cells. Altogether, these data suggested that NOTCH activation promoted the recruitment of cardiac non-myocyte cells (NMCs) into the cardiogenic lineage but disallowed CM differentiation. In this vein, inhibition of NOTCH signaling has been shown to favor cardiac transcription factor-mediated reprogramming of fibroblasts into induced CPCs and CMs [29]. These observations suggest that a certain degree of plasticity exists in the cardiac non-myocyte population, which could be exploited to convert a priori non-cardiogenic cells into CMs via manipulating the NOTCH pathway. Furthermore, if true, CM production might occur at the expense of myofibroblast differentiation. In the present study, we therefore examined the possibility that inhibition of NOTCH1 or NOTCH2 signaling in vivo in a situation of cardiac hypertrophy may facilitate the emergence of CMs from non-myocyte origins.

## 2. Materials and Methods

### 2.1. Animals

The Government Veterinary Office (Lausanne, Switzerland) approved animal experiments that were performed according to the University of Lausanne Medical School institutional guidelines and the guidelines from Directive 2010/63/EU of the European Parliament. *Myh6-Jagged1* (TGJ1) mice were generated in this laboratory and were described previously [28]. *N1IP-CreErt2* mice (IMSR Cat# JAX:006953, RRID:IMSR_JAX:006953) [30] were obtained from R. Kopan; *ROSA26-mTmG* mice (IMSR Cat# JAX:007676, RRID:IMSR_JAX:007676) [31] were obtained from Dr L. Luo and *Myh6-MerCreMer* mice (IMSR Cat# JAX:005657, RRID:IMSR_JAX:005657) [32] were obtained from Dr J. Molkentin. C57bl/6J (IMSR Cat# JAX:000664, RRID:IMSR_JAX:000664) mice were purchased from Charles River (Ecully, France).

### 2.2. Lineage Tracing

The dual fluorescence reporter mouse line *ROSA26-mTmG* [31] was crossed to *N1IP-CreErt2* [30] or to *Myh6-MerCreMer* [32] mouse lines in order to genetically label cells experiencing NOTCH1 signaling or CMs, respectively. The conversion from tdTomato to EGFP expression with the *N1IPCreERT2* reporter line was induced by intraperitoneal injection of Tamoxifen 2 days before, 2 days after, and on the day of surgery, at a dose of 67 μg/g body weight. For labeling of CMs using the *Myh6-MerCreMer* line, Tamoxifen was injected during five consecutive days and surgery was performed after 1 week of recovery.

### 2.3. Surgery and Echocardiography

Transaortic constriction was performed as described previously [28]. Briefly, 12–14-week mice were anesthetized by intraperitoneal injection of ketamin/xylazine/acepromazin (65/15/2 mg/kg body weight). Body temperature was maintained by placing mice on a warmed pad. Transaortic constriction (TAC) was performed using a 7.0 silk suture tied twice around the aorta between the right innominate artery and the left common carotid and a predetermined-gauge needle size (25 gauge). The needle was then gently retracted leaving a restriction of the same caliber among different mice. For animals undergoing a sham operation, the ligature was placed in an identical location but was not tied. Trans-thoracic echocardiography was performed using a 30 MHz probe and the Vevo 770 Ultrasound machine (Visualsonics, Toronto, ON, Canada) under mild anesthesia with 1–1.5% Isoflurane. Diastolic and systolic internal ventricular septum (IVS;d and IVS;s), diastolic and systolic left ventricular free posterior wall thickness (LVPW;d and LVPW;s), and left ventricular internal end-diastolic and end-systolic chamber (LVID;d and LVID;s) dimensions were measured three times on M-mode images. Left ventricular fractional shortening (%FS) and ejection fraction (%EF) were calculated. The right ventricle internal diameter and wall thickness were also measured on parasternal short axis view, using papillary muscles as reference points. Transaortic velocity was determined using the Doppler mode.

### 2.4. Antibody, BrdU and GapmeR Administration

Antibodies against the NOTCH1 and NOTCH2 negative regulatory regions (αNRR1 and αNRR2, respectively) were injected IP, twice, four days apart, at a dose of 5 mg/kg as described [18]. The antibodies were administered 2 weeks after surgery, unless otherwise indicated. Control mice received vehicle (PBS, Boston, MA, USA). BrdU (Sigma-Aldrich, St. Louis, MO, USA) was administered to mice as a 10 mg/mL solution in drinking water supplemented with 1.8% sucrose. Control and *Wisper* antisense LNA GapmeR design and administration was described previously [15]. Briefly, Control GapmeRs and Wisper-GapmeR (Qiagen, Hombrechtikon, Switzerland) were diluted in NaCl 0.9% isotonic solution to obtain the final working concentration (5 mg/kg) just before the injection via intraperitoneal route.

### 2.5. RNA Isolation and Quantitative RT-PCR Analysis

RNA was isolated from mouse tissues using Trizol reagent according to the manufacturer’s instructions (Thermo Fisher, Waltham, MA, USA). Total RNA (4 μg) was reverse-transcribed using MMLV reverse transcriptase (Invitrogen, Waltham, MA, USA) and oligodT primers (Microsynth, Balgach, Switzerland) followed by quantitative TaqMan RT-PCR analysis using Gene Expression Assays (Applied Biosystems, Waltham, MA, USA, Cat# 4331182) or SYBR green qPCR and custom-made gene-specific primers (Supplementary Table S1). Amplification was performed on Life Technologies QuantStudio 6 or RT-PCR System, (RRID:SCR_020239) or Applied Biosystems QuantStudio 12 K Flex RT-PCR System (RRID:SCR_021098). Expression data were normalized to Gapdh and relative expression levels were calculated according to the −ΔΔCt method, and expressed as fold change relative to reference samples, as indicated in individual experiments.

### 2.6. Immunofluorescence Staining

Immunostaining was performed as described previously [28,33] using the antibodies listed in Supplementary Table S1. For BrdU detection, frozen heart tissue sections were thawed and fixed in 2% paraformaldehyde (PFA) in PBS for 20 min at room temperature. DNA was fragmented by incubation in 2N HCl at 37 °C for 20 min followed by neutralization in 0.1M Borate buffer, pH 8.5. BrdU detection was performed using rat anti-BrdU antibody and the appropriate Alexa fluorochrome-conjugated anti-rat antibody. For the co-staining with BrdU and other antigens, tissue sections were first subjected to the complete staining procedure to detect the desired protein antigens. Tissue sections were then post-fixed with 2% PFA for 10 min at room temperature, and then processed for BrdU detection. For quantitative analysis of BrdU incorporation, fluorescence photomicrographs were taken at 40× magnification, and the cells were counted manually on the micrographs. To unambiguously identify BrdU-incorporating CMs, heart tissue sections were labelled with antibodies against BrdU, sarcomeric α-actinin and laminin; nuclei were stained with DAPI. A CM was scored as BrdU+ if it displayed nuclear BrdU staining immediately surrounded by α-actinin signal within boundaries marked by laminin. Because CM nuclei were not always visible, depending on the section plan, the BrdU labelling index is expressed as percent of CMs with visible nuclei. Where applicable, DAPI-stained nuclei were counted using ImageJ software. A minimum of 5 micrographs were taken for each heart tissue section. For estimation of the number of tdT+ CMs, heart tissue sections were stained with antibodies against EGFP and α-actinin. For quantification, tiled fluorescence micrographs were taken at 10 or 20× magnification using Zeiss Axiovision and the Stitch function of the software (AxioVision Imaging System, Carl Zeiss, Feldbach, Switzerland, RRID:SCR_002677). Alternatively, images were acquired using a Zeiss AxioScan and processed using the Zen2 Software. The stitched images were adjusted for brightness and contrast using Adobe Photoshop. Cell counts of tdT+ and α-actinin+ CMs were performed manually on the stitched micrographs. Tissue sectional area was determined using ImageJ software and the total number of CMs per section was calculated from known numbers of CMs per area unit counted from random micrographs taken at 40× magnification.

### 2.7. Isolation of CMs and Non-Myocyte Cells from Adult Mouse Hearts

Hearts were washed by retrograde perfusion through the aorta using cannulas hooked to 1.0 mL syringes. Individual hearts were washed with 5.0 mL HBSS solution then fixed with 3.0 mL of 4% PFA in PBS during 5 min. The hearts were washed again with 3.0 mL of HBSS and finally rinsed with 1.0 mL of enzyme solution (Collagenase type 2, Worthington; 4 mg/mL and Protease IV, Roche; 1 mg/mL in HBSS) and kept on ice until all hearts have been harvested. The cannulated hearts were then digested 3 times at 37 °C, 15 min each, with a change of digestion solution at each digestion cycle. The digested hearts were cleared of atria and blood vessels, and the ventricles were teased into small pieces with forceps and triturated with a wide-bore pipette. Undigested fragments were left to sediment and the supernatants containing single-cell suspensions were centrifuged at 18× *g* for 3 min to pellet CMs. For BrdU immunostaining, CM preparations were fixed for 10 min at room temperature in 4% PFA, washed and resuspended in PBS. Aliquots containing approximately 5 × 10^4^ isolated CMs were immobilized on microscope slides using Cell Tak reagent (Corning). After drying, the attached cells were permeabilized with 0.25% Triton X-100 and treated with 2.5 Units of DNase I per slide (New England Biolabs, Ipswich, MA, USA), and stained for BrdU and α-actinin as described for tissue sections.

### 2.8. Histology and Fibrosis Measurements

Hearts were fixed in 4% formalin overnight. Paraffin tissue sections were processed for hematoxylin and eosin or Masson Trichrome staining using standard histological procedures. The determination of percent fibrotic areas was performed by measuring collagen deposition (blue) on micrographs of Masson Trichrome-stained slides using the NIH ImageJ software. Quantification was performed on whole heart tissue sections. The 8-bit color micrographs were converted to RGB stacks, and threshold adjusted to the channel showing the highest contrast matching the blue color on the original image. The total cross-sectional area was also measured. Amount of fibrosis was determined by calculating the percentage of fibrosis area relative to total area.

### 2.9. Transfection of P19Cl6 Cells and Activation of Wisper Expression

P19CL6 cells (RCB Cat# RCB2318, RRID:CVCL_L981) were cultured in Dulbecco’s modified Eagle’s medium with 10% fetal calf serum (FCS; Life Technologies, Carlsbad, CA, USA). To activate *Wisper* lncRNA expression using the CRISPR-based Synergistic Activation Mediator (SAM) system, the cells were co-transfected with pGAG-SAM (expressing dCAs9:vp64;T2A;MS2-p65-HSF1, SAM components, under CAG promoter) and a *Wisper* targeting guide RNA consisting of the sgRNA (MS2) cloning backbone (Addgene plasmid no. 61424) in which the *Wisper*-targeting sequence GTCGACTCTGCTATACTCCA was inserted [sgRNA(MS2)-*Wisper*-sg]. Plasmids were mixed at a 1:1 ratio and transfected using Lipofectamine 2000 reagent (Life Technologies) according to the manufacturer’s instructions. Total cellular RNA was isolated 48 h later using the miRNeasy kit (Qiagen, Hombrechtikon, Switzerland) and subjected to qRT-PCR analysis.

### 2.10. Statistical Analysis

One way ANOVA Multiple Comparisons Test was used to assess significance of differences between experimental groups. For comparison of two groups, two-sided, unpaired student *t*-test was used. Data throughout this paper are presented as Boxplots showing Min, Max and Median values or Bar graphs showing Mean ± SEM. *p* values < 0.05 were considered as significant. Analysis was performed using GraphPad Prism version 8.3.0 (GraphPad Prism, San Diego, CA, USA, RRID:SCR_002798).

## 3. Results

### 3.1. The NOTCH Signaling Pathway Is Activated in Cardiac Non-Myocyte Cells Isolated from the Stressed Heart

To evaluate the contribution of the NOTCH pathway during the response of the adult heart to stress, we took advantage of transgenic mice in which Jagged1 was under control by the Myosin heavy chain (Myh)6 promoter (TGJ1 mice) and therefore overexpressed in CMs (28). We have shown previously that NOTCH receptor activation takes place between Jagged1-overexpressing CMs and adjacent CMs and NMCs during pressure overload, and that NOTCH signaling was enhanced in TGJ1 mice. To further assess the NOTCH response to pressure overload, we isolated NMCs (comprising in particular cardiac fibroblasts, CPCs and endothelial cells) and CMs from adult mouse hearts one week after TAC (Supplementary Figure S1A). NMCs were characterized by an increased expression of fibrosis markers in TAC-operated wild-type (WT) and TGJ1, relative to Sham-operated mice (Supplementary Figure S1B). We next measured the expression of NOTCH receptors and ligands, as well as of NOTCH target genes (Supplementary Figure S1C). In WT mice, TAC induced the expression of all components of the NOTCH pathway. This was also the case in TGJ1 mice. In addition, as expected, Jagged1 was more expressed in TGJ1 mice as compared to WT, both under basal conditions (Sham) and under stress (TAC). In turn, we observed that Notch1 and Notch2 expression was also higher in transgenic mice. Globally, the response of the NOTCH pathway was increased in TGJ1 mice as judged in particular by target gene expression. Contrary to NMCs, no significant induction of NOTCH-related genes was measured in CMs (Supplementary Figure S1C).

### 3.2. NOTCH1 Blockade Inhibits Pressure Overload-Induced Hypertrophy and Preserves Cardiac Function

**Figure 1:**
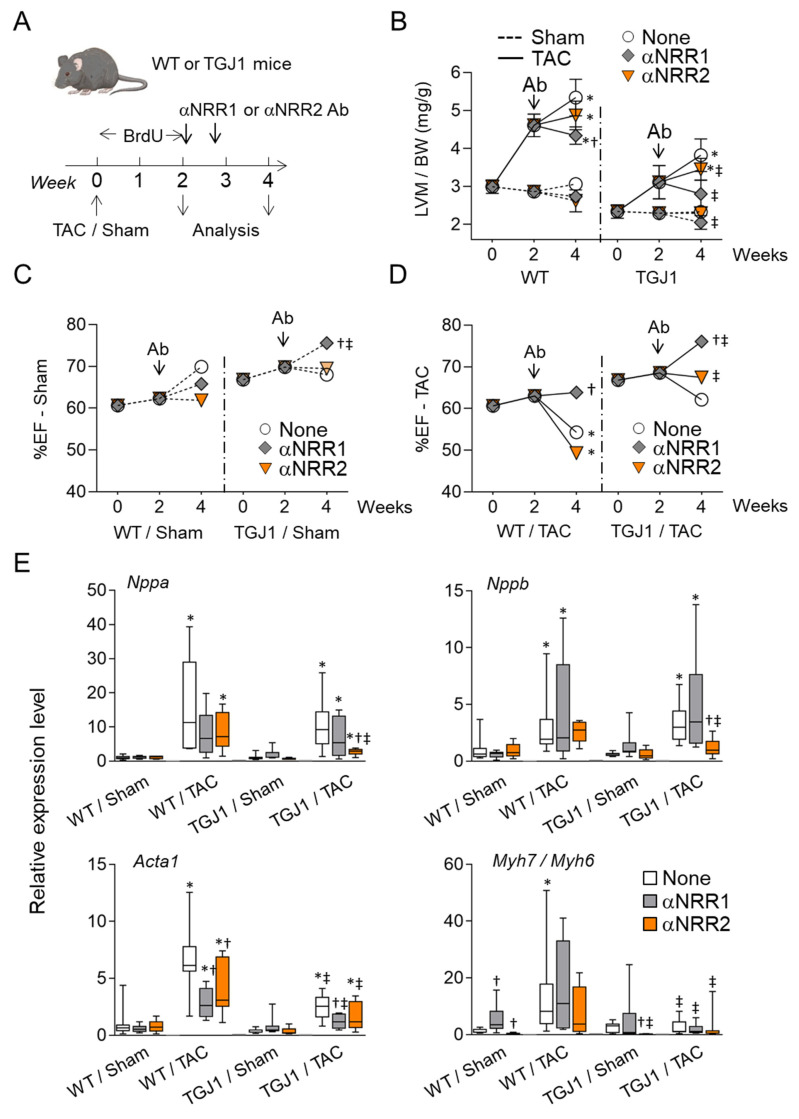
NOTCH1 blocking antibody treatment attenuates cardiac hypertrophy. (**A**) Experimental protocol. TGJ1 mice and WT controls were subjected to Sham or transaortic constriction for two weeks, then treated twice with αNRR1 or αNRR2 antibodies (Ab) at 4 days interval. Untreated mice (none) were also included. BrdU was administered during the first two weeks to label proliferating cells. Hearts were harvested 4 weeks after TAC and analyzed. (**B**) Left ventricle mass-to-body weight ratio in mg/g measured at baseline and week 2 and 4 post surgery. (**C**,**D**) Progression of percent ejection fraction (%EF) from baseline to week 4, in Sham (**C**) or TAC (**D**) operated mice. (**E**) RT-PCR analysis of expression of cardiac stress marker genes Acta1, Nppa, Nppb and Myh7/Myh6 ratio. The graphs represent expression values relative to untreated, Sham-operated group; *n* = 5–10 animals/group (see Supplementary Table S2). *, *p* < 0.05 in TAC vs. Sham; †, *p* < 0.05 in αNRR vs. None; ‡, *p* < 0.05 in TGJ1 vs. WT.

We previously evaluated the response of TGJ1 mice to pressure overload. These mice demonstrated reduced myofibroblast differentiation and increased CPC production [28]. Nevertheless, CPCs failed to further differentiate into CMs under these conditions. Since NOTCH is known as an inhibitor of cardiac differentiation when chronically activated, we examined the effects of NOTCH1 and NOTCH2 blockade on the cardiac response to pressure overload in WT and TGJ1 mice. Mice were subjected to TAC, and 2 weeks thereafter received anti-NRR1 (αNRR1) or anti-NRR2 (αNRR2) antibodies, targeting NOTCH1 or NOTCH2 receptors, respectively. Then, functional analysis using echocardiography was performed after another two-week period (Figure 1A). Four weeks after TAC, control WT hearts displayed a typical hypertrophic response characterized by an increase in left ventricle mass-to-body weight ratio (LVM/BW; Figure 1B). As expected, cardiac hypertrophy was significantly reduced in TGJ1 mice (Figure 1B and Supplementary Table S2). We next compared animals administered anti-NOTCH receptor antibodies. Anti-NRR1 treatment significantly attenuated TAC-induced hypertrophy in both WT and TGJ1 mice. However, this was not the case when mice received αNRR2 antibodies (Figure 1B and Supplementary Table S2). Under prolonged pressure overload, the myocardium undergoes adverse remodeling, which leads to diminished cardiac function. Compared to sham-operated animals, ejection fraction (%EF) was significantly lower in untreated WT mice 4 weeks after TAC. Interestingly, deterioration of function was abolished in mice treated with αNRR1 antibodies while no effects of αNRR2 antibodies were observed. Moreover, function, which was already preserved in untreated TGJ1 mice as compared to WT animals, was further enhanced in transgenic mice receiving αNRR1 antibodies (Figure 1C,D and Supplementary Table S2). Again, αNRR2 antibodies had no impact on cardiac function under these conditions. We next measured expression of cardiac markers of stress. Globally, αNRR1 antibodies, and to a greater extent αNRR2 antibodies, attenuated the induction of stress genes after TAC, an effect that was more pronounced in TGJ1 mice than in WT mice (Figure 1E).

### 3.3. NOTCH1 Blockade Reduces Cardiac Fibrosis in the Stressed Heart

**Figure 2:**
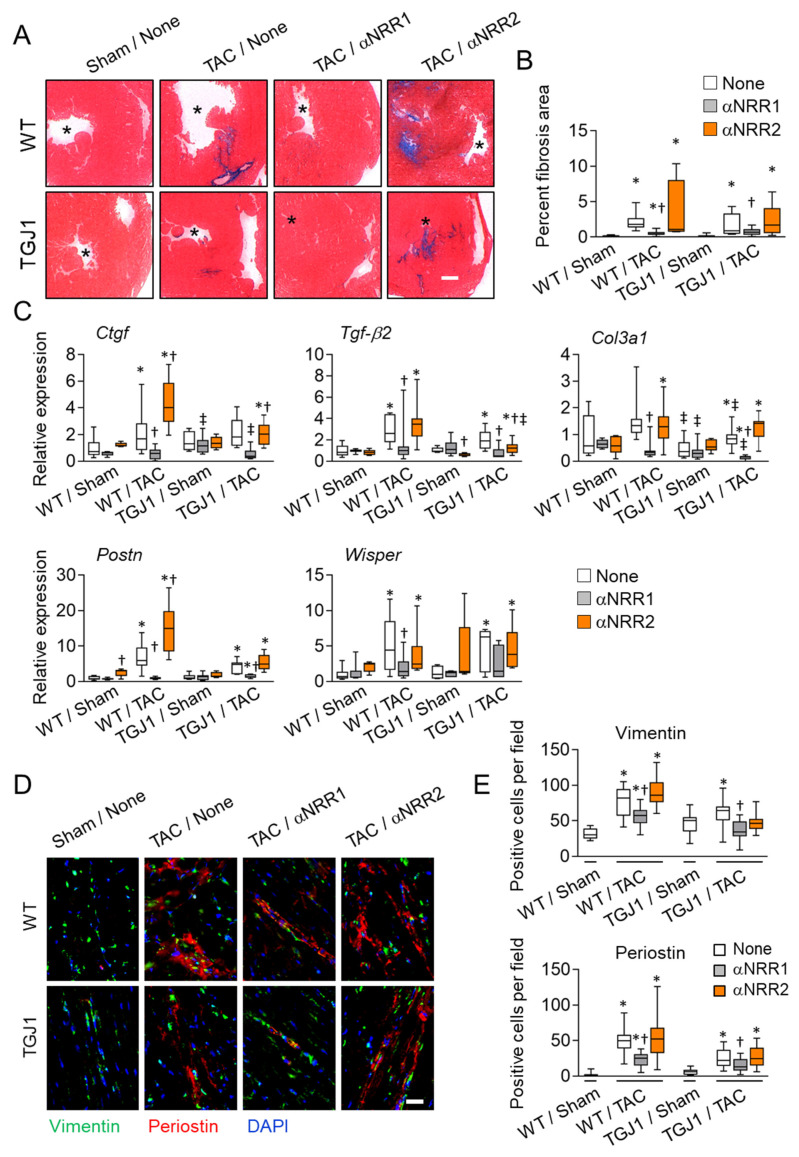
NOTCH1 and NOTCH2 antibody blockade exerts different effects on cardiac fibrosis. (**A**) Mice were operated as in Figure 1, and transverse heart tissue sections were subjected to histological Masson Trichrome staining to reveal collagen deposition (blue). Asterisks indicate the left ventricle cavity. (**B**) Interstitial fibrosis was measured on heart tissue sections stained as in (**A**) and expressed as percent of total heart section area. (**C**) RT-PCR analysis of expression of fibrosis marker genes Ctgf, Tgf-β2, Col3a1, Postn and Wisper in control WT and TGJ1 mice subjected to sham or TAC operation with αNRR1 or αNRR2 antibody or without antibody treatment (None). Expression levels are relative to untreated, Sham-operated group. (**D**,**E**) Immunofluorescence staining of heart tissue sections using anti-vimentin (green) and anti-periostin (red) antibodies. *n* = 5–10 animals/group (see Supplementary Table S2). *, *p* < 0.05 in TAC vs. Sham; †, *p* < 0.05 in αNRR vs. None; ‡, *p* < 0.05 in TGJ1 vs. WT. Scale bar in B = 200 μm, in (**D**) = 20 μm.

Cardiac fibrosis develops in hearts subjected to chronic pressure overload. Indeed, cardiac tissues were characterized by increased interstitial and peri-vascular fibrosis after TAC (Figure 2A,B). Importantly, the development of fibrosis was abolished in hearts of mice administered αNRR1 antibodies. In contrast, the αNRR2 treatment had no effects on fibrosis. The fibrotic response was further attenuated in TGJ1 mice. In agreement, expression of fibrosis markers, such as Ctgf, Tgfβ2, Postn, Col3a1 and Wisper, was induced in the stressed WT heart, and to a lower extent in TGJ1 mice. Induction was reduced by administration of αNRR1 antibodies but not following αNRR2 injection (Figure 2B,C). We also quantified the numbers of POSTN+ and VIM+ activated fibroblasts, i.e., myofibroblasts, in fibrotic lesions. Accumulation of myofibroblasts was a characteristic feature of the stressed hearts, and was attenuated by αNRR1 treatment (Figure 2D,E). Anti-NRR2 antibodies did not reduce myofibroblast differentiation. Once again, the global response was lower in TGJ1 mice. Overall, these data suggested that fibrosis was a clear determinant of cardiac dysfunction, and that the beneficial effects associated with αNRR1 treatment resulted primarily from its anti-fibrotic action, something that was not seen following αNRR2 administration. In this regard, we evaluated expression of NOTCH receptors, ligands and target genes in NMCs isolated from the stressed heart following either NOTCH1 or NOTCH2 inhibition (Supplementary Figure S2). Anti-NRR2, but not αNRR1, treatment substantially increased the level of NOTCH receptor expression in the hearts of both WT and TGJ1 mice.

**Figure 3:**
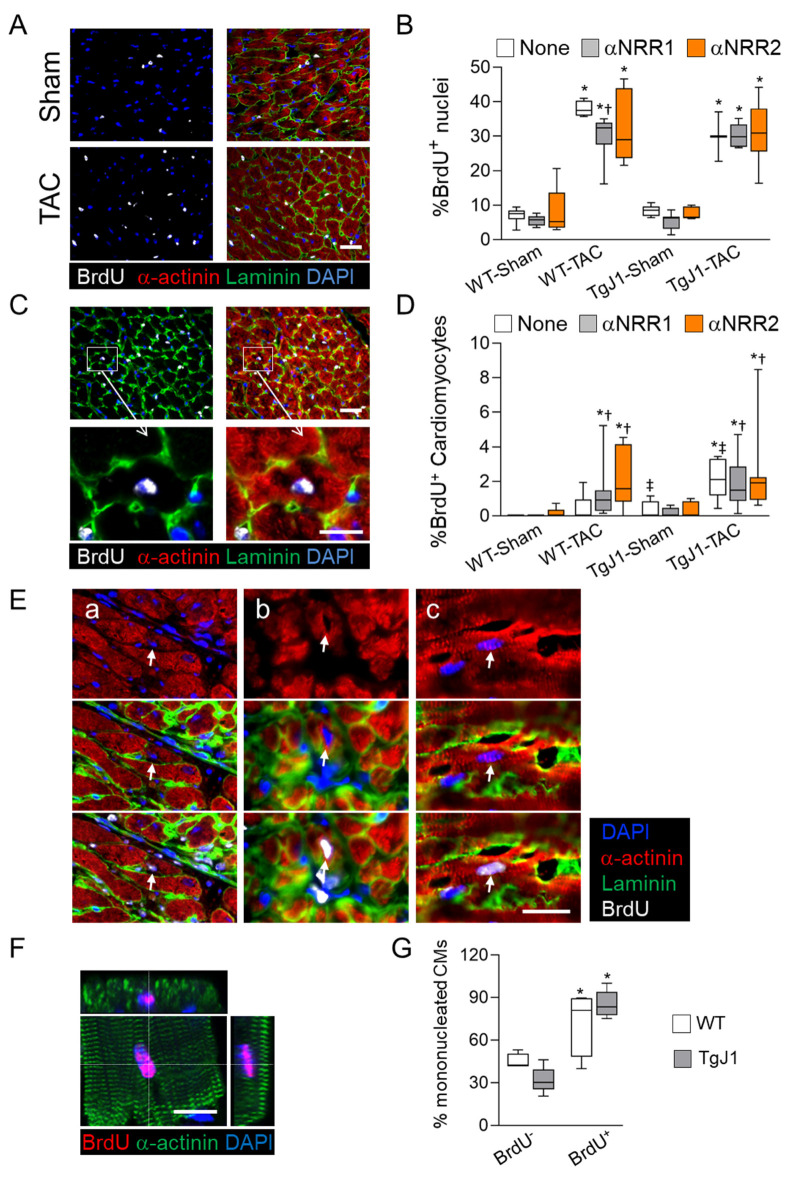
Aortic constriction increased BrdU incorporation in the myocardium. Mice were subjected to Sham or TAC operation as in Figure 1A and heart tissue sections were subjected to immunostaining to reveal BrdU incorporation. (**A**) Micrographs showing tissue sections stained with antibodies against BrdU (Gray), α-actinin (red), Laminin (green) and DAPI (blue). (**B**) Quantitative analysis of total BrdU incorporation expressed as percentage of BrdU+ nuclei. (**C**) Examples of BrdU+ CMs in the myocardium, identified by virtue of BrdU+ nuclei within sarcomeric α-actinin+ cytoplasm delimited by a laminin+ signal to mark CM boundaries. The insets show the BrdU+ CMs at high magnifications. (**D**) Quantification of BrdU+ CMs. The results are expressed as percent BrdU+ CMs relative to total CMs with visible nuclei. (**E**) Examples of small BrdU+ CMs. The micrographs show small *α*-actinin+ and BrdU+ CMs (arrows) with faint laminin extracellular matrix organization in sub-endocardial regions (**a**), in proximity of blood vessels (**b**), and in the myocardium (**c**). (**F**,**G**) Single CMs isolated from WT and TGJ1 mouse hearts subjected to TAC and αNRR1 treatment were immunostained using antibodies against BrdU (red) and α-actinin (green) and DAPI. (**F**) Confocal image of a BrdU+ CM isolated from adult heart with orthogonal views showing BrdU+ nucleus within α-actinin-stained sarcomeres (**G**) The percentage of mono-nucleated BrdU+ (pos.) and BrdU− (neg.) CMs were determined on CMs stained as in (**E**). (**B**,**D**) *n* = 5–10 mice/group (see Supplementary Table S2); for (**F**), WT, *n* = 4; TGJ1, *n* = 5. *, *p* < 0.05 in TAC vs. Sham; †, *p* < 0.05 in αNRR vs. None; ‡, *p* < 0.05 in TGJ1 vs. WT. Scale bars = 20 μm.

Pressure overload induces the proliferation of cardiac NMCs. Indeed, substantial BrdU incorporation was measured throughout the myocardium after 4 weeks of TAC relative to sham-operated animals (Figure 3A,B). No significant difference was observed in global BrdU incorporation between untreated mice and those receiving αNRR1 or αNRR2 treatment. We therefore examined whether NOTCH1 or NOTCH2 blockade could increase BrdU incorporation in α-actinin+ CMs (Figure 3C). BrdU+ CMs were rare or undetected in sham-operated WT mouse hearts and represented only 0.07% of CMs after TAC (Figure 3D). Interestingly, the percentage increased to 0.32% and 0.59% of CMs in sham-operated and TAC TGJ1 transgenics, respectively. Anti-NRR1 antibody increased the percentage of BrdU+ CMs in both WT and TGJ1 hearts after TAC to 0.72% and 1.12%, respectively. In the αNRR2-treated mice, the percentage of BrdU+ CMs increased even to 2.18% in WT and 2.43% in TGJ1mice. The BrdU+ CMs were detected throughout the myocardium, in sub-endocardial regions, in areas of intense remodeling and in proximity of blood vessels, where the small α-actinin+ and BrdU+ CMs appeared small, immature and with a less-organized laminin+ extracellular matrix (Figure 3E(a–c)). Cell cycle activity in adult CMs can lead to cell division or bi-nucleation [34,35]. Therefore, we analyzed BrdU incorporation and nucleation in single CMs isolated from hearts subjected to TAC and αNRR1 treatment in both WT and TGJ1 mice (Figure 3F,G). The data showed that BrdU+ CMs were nearly 80% mono-nucleated, whereas BrdU-negative CMs were only 30–40% mono-nucleated. These data suggested that the BrdU+ CMs might arise either from the division of rare immature CMs or from the differentiation of undifferentiated CPCs recruited into the CM fate under pressure overload and NOTCH1 inhibition.

### 3.4. NOTCH1-Activated Cells Contribute to Non-Myocyte Cell Formation

NOTCH1, but not NOTCH2 blockade, blunted the development of cardiac hypertrophy and fibrosis, and preserved cardiac function in mice subjected to pressure overload. Therefore, we sought to determine the progeny of NOTCH1-activated cells. For this purpose, we crossed *N1IP-CreErt2* mice [30] expressing a NOTCH1 intracellular domain (NOTCH1-IC), Tamoxifen-inducible, Cre fusion protein to *ROSA26-mTmG* mice [31], to generate double transgenic mice (hereafter referred to as NOTCH1 reporter mice). In the presence of Tamoxifen, a switch from tdTomato (tdT) to EGFP expression occurs in cells subjected to NOTCH1 signaling (Supplementary Figure S3A). To test the efficacy of the αNRR1 treatment under these conditions, reporter mice received blocking antibodies before initiating Tamoxifen-induced tdT-to-EGFP conversion (Supplementary Figure S3B). As expected, the EGFP signal was abolished in mice pre-treated with αNRR1 antibodies to block NOTCH1 receptor, formally demonstrating the capacity of the antibodies to terminate a NOTCH1 signal in the heart.

**Figure 4:**
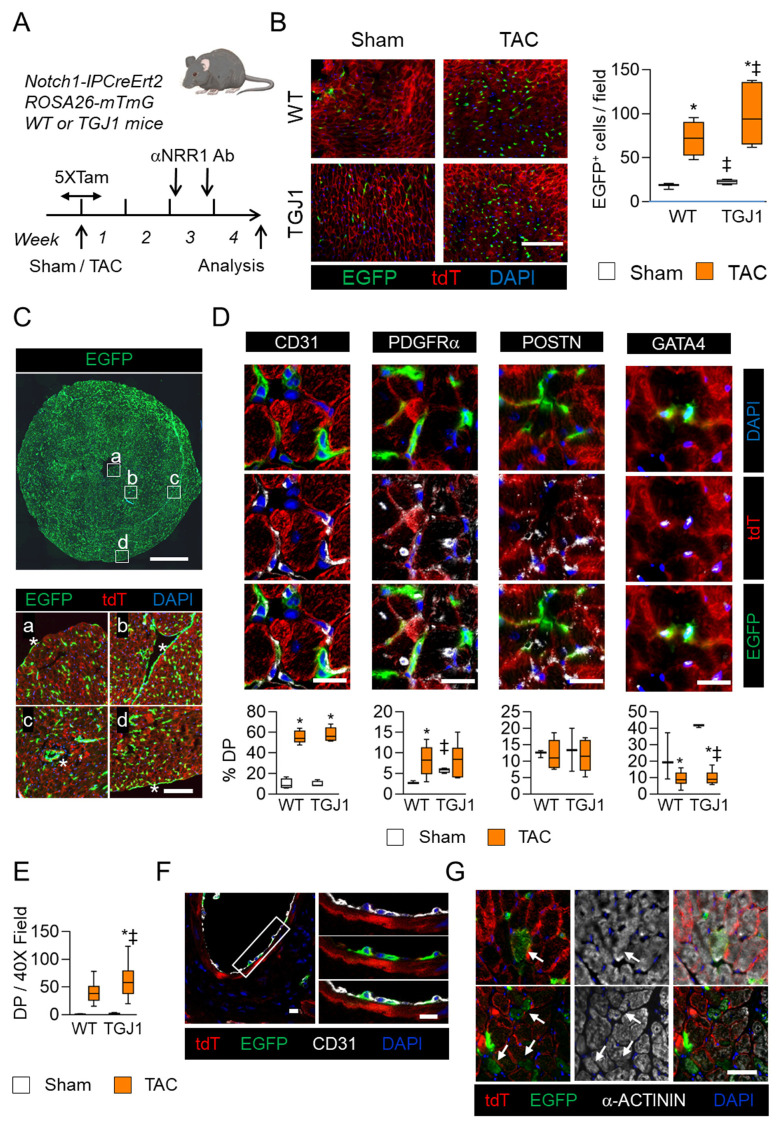
Fate of NOTCH1-activated cells in the stressed heart. (**A**) Experimental protocol. Wild type and TGJ1 mice harboring Notch1-IPCreErt2 and ROSA26-mTmG alleles were subjected to TAC or Sham operation. Mice were injected with Tamoxifen to induce tdT to EGFP conversion in cells experiencing NOTCH1 signaling in the 2 days before, the 2 days after and on the day of surgery. After 2 weeks, they were injected with αNRR1 antibody. Analysis was performed 4 weeks after operation. (**B**) Photomicrograph of heart tissue sections showing NOTCH1-traced EGFP+ cells in the myocardium of Sham and TAC-operated αNRR1-treated WT and TGJ1 mice. The graph on the right shows the number of EGFP+ cells per 40X microscope field. (**C**) Whole heart tissue section showing NOTCH1-traced cells. The insets marked (**a**–**d**) show NOTCH1-traced cells in the endocardium of the left and right ventricles (**a**,**b**), in a small blood vessel (**c**) and in the epicardium (**d**), which are indicated by asterisks. (**D**) Characterization of NOTCH1-traced EGFP+ cells. Tissue sections were stained with anti-EGFP (green) and with either anti-CD31, anti-PDGFRα, anti-PERIOSTIN or anti-GATA4 antibodies (Gray); non-traced cells express tdT (red). The graphs represent the percentage of double positive (% DP) EGFP+ cells expressing the indicated markers in WT and TGJ1 mice subjected to Sham or TAC surgery and αNRR1 treatment. (**E**) Number of CD31+-EGFP+ double-positive (DP) cells per 40X field in WT and TGJ1 mice subjected to Sham or TAC operation. (**F**) NOTCH1 traced cells in the endothelium of a large blood vessel. Heart tissue sections were stained for CD31 (Gray) and EGFP to reveal NOTCH1-traced cells. The insets show the area demarcated by the rectangle at high magnification. (**G**) Fluorescence micrographs showing NOTCH1-traced EGFP+ CMs (arrows), identified as single CMs or clusters of several CMs. The EGFP+ CMs (Green) also express α-actinin (Gray), as do the adjacent tdT+ CMs (Red). In (**B**,**D**,**E**,**F**), WT-Sham, *n* = 3; TGJ1-Sham, *n* = 4; WT-TAC, *n* = 6; TGJ1-TAC, *n* = 5. *, *p* < 0.05 in TAC vs. Sham; ‡, *p* < 0.05 in TGJ1 vs. WT. Scale bar in (**B**) = 200 μm; in (**C**) = 1 mm; in insets (**a**–**d**) = 100 μm; in (**D**,**G**) = 50 μm.

We next addressed the fate of NOTCH1-activated cells in the stressed heart of WT and TGJ1 mice following NOTCH1 antibody administration. WT control, i.e., N1IP-CreErt2; ROSA26-mTmG, and TGJ1 NOTCH1 reporter mice, i.e., Myh6-Jagged1; N1IP-CreErt2; ROSA26-mTmG, underwent TAC surgery. Tamoxifen was administered during five consecutive days to label NOTCH1-activated cells around the time of surgery (Figure 4A). Two weeks after TAC, mice were injected twice with αNRR1 antibodies, and then analyzed two weeks later. The total number of NOTCH1-traced EGFP+ cells increased in TAC relative to sham-operated hearts (Figure 4B). EGFP+ cells were significantly more abundant in TGJ1 hearts relative to WT. Spatially, EGFP+ cells were found in the endocardium, the myocardium and the epicardium (Figure 4C).

EGFP+ NOTCH1-traced cells were for the most part CD31+ endothelial cells located in capillaries and in small and large blood vessels (Figure 4D–F). Accordingly, the numbers of CD31+/EGFP+ cells were found to be greater in TGJ1 hearts as compared to WT controls (Figure 4E). Capillary density also appeared higher in TGJ1 mice after TAC, although not significantly different (Supplementary Figure S3C). Importantly, PDGFRα+/EGFP+ cells, known precursors of endothelial cells, were also found increased upon pressure overload (Figure 4D). Although the percentages were not different in WT and TGJ1 mice after TAC, PDGFRα+ cells were found in larger amounts in TGJ1 transgenics under basal conditions. In contrast, no differences were observed in the percentages of POSTN+/EGFP+ cells during these various manipulations. Finally, a subset of EGFP+ cells expressed the early cardiac marker GATA4, indicating a possible commitment to the cardiac lineage. Interestingly, the percentages of GATA+/EGFP+ cells decreased during the response of the heart to stress, suggesting potential recruitment of the cells during adaptation to pressure overload. Thus, we searched for EGFP+ CMs. Careful observation indicated that EGFP+ CMs were eventually found, but were extremely rare (Figure 4G). Only, six out of eleven mice showed a few EGFP+ CMs per heart section. Clusters of three to five small, α-actinin+/EGFP+ CMs were also observed. This indicated that, although manipulating the NOTCH pathway promoted CM formation, these cells were not direct progeny of NOTCH1-expressing precursors.

### 3.5. NOTCH Blockade Stimulates CM Formation from Non-Myocyte Cells in the Stressed Heart

To determine whether new CMs were produced from NMCs following sequential activation and blockade of the NOTCH pathway, we used Myh6-MerCreMer; ROSA26-mTmG mice [31,32]. In these mice, Tamoxifen-activated recombination of the ROSA26-mTmG locus leads to a switch from tdTomato (tdT) to EGFP expression in CMs. After Tamoxifen administration, preexisting CMs and their progeny express EGFP. By contrast, NMCs and their progeny express tdT (Supplementary Figure S4A,B). Importantly, highly efficient conversion of EGFP-expressing cardiomyocytes into tdT-expressing cells occurs in more than 99% of the cardiomyocyte population (Supplementary Figure S4C).

**Figure 5:**
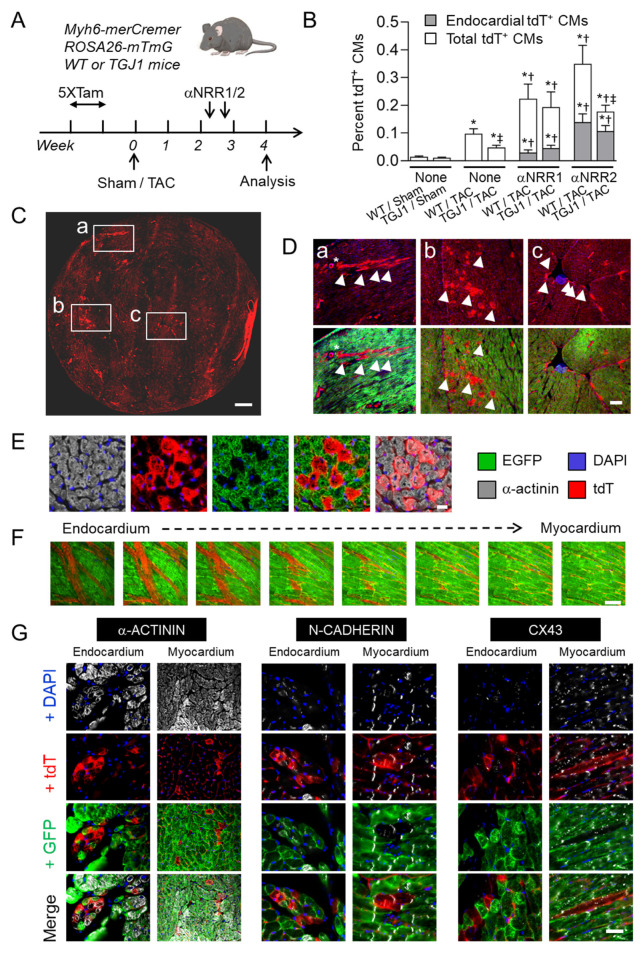
New myocytes originate from non-myocyte cells. (**A**) Experimental protocol. TGJ1 and WT control mice bearing Myh6- MerCreMer and ROSA26-mTmG transgenes were injected with Tamoxifen during 5 consecutive days to induce a conversion from tdT to EGFP expression specifically in CMs. The mice were subjected to Sham or TAC operation. After two weeks, the mice were injected twice with αNRR1 or αNRR2 antibody at four days interval. Mice were sacrificed and analyzed after 4 weeks of TAC. (**B**) Quantification of new tdT+ CMs in WT and TGJ1 mice subjected to Sham or TAC operation and treated with αNRR1, αNRR2 antibodies or none. The data show percentage of tdT+ CMs per heart section. The proportions of tdT+ CMs in the endocardium region are shown in Gray. *, *p* < 0.05 in TAC vs. Sham; †, *p* < 0.05 in αNRR vs. None; ‡, *p* < 0.05 in TGJ1 vs. WT (*n* = 4–8 mice per group). (**C**) Representative whole heart tissue section showing the three main locations of tdT+ CMs (Red). The rectangles denote: (**a**) a perivascular region, (**b**), a ventricular myocardium region and (**c**) a endocardial region. The tdT+ CMs (arrowheads) in the three different localizations are shown at higher magnification in (**D**). (**E**) Cluster of tdT+ (Red) α-actinin+ (Gray) CMs and adjacent pre-existing EGFP+ (Green) CMs (**F**). Wholemount confocal serial sections in the endocardium to myocardium axis showing tdT+ CMs (Red) as a one-cell deep layer. (**G**) Characterization of the tdT+ CMs. Heart tissue sections were stained (Gray) with antibodies against α-actinin, to mark CM cytoplasm, N-Cadherin to mark intercalated disks, and against Connexin-43 to label gap junctions. Representative areas with new tdT+ CMs (Red) are shown, in the myocardium and in the subendocardium, adjacent to pre-existing CMs (Green). Scale bars in (**C**) = 0.5 mm, in (**D**) = 200 μm (right) and in (**E**–**G**) = 50 μm.

Tamoxifen-treated mice were then subjected to TAC, and administered either αNRR1 or αNRR2 antibodies two weeks thereafter (Figure 5A). Mice were analyzed two weeks following antibody administration by immunohistochemistry (Figure 5B–G). In sham-operated mice, the percentage of tdT+ α-actinin+ CMs represented only 0.01% of the total population in both WT and TGJ1 mice whereas in untreated TAC mice, the percentage of tdT+ CMs increased to 0.1% and 0.05% respectively. We next analyzed TAC mice treated with αNRR1 or αNRR2 antibodies. In αNRR1-treated mice, the percentages of tdT+ CMs represented 0.22% in WT controls and 0.19% of the total CM population in TGJ1 transgenics. In αNRR2-treated mice, these percentages increased to 0.35% and 0.18% in WT and TGJ1 mice, respectively. This represents therefore a substantial increase in the number of tdT+ α-actinin+ CMs as compared to controls.

Spatially, tdT+ and α-actinin+ CMs were found as clusters throughout the myocardium. In particular, important clusters were present in the ventricular walls adjacent to large blood vessels and directly in the subendocardial region (Figure 5C–F). Of note, no overlap between tdT and EGFP fluorescence was observed, making it unlikely that the tdT+ CMs resulted from fusion of tdT+ NMCs with pre-existing EGFP+ CMs (Figure 5E). Quantification revealed that subendocardial tdT+ CMs represented a significant portion of newly formed CMs following anti-NOTCH treatment in TAC mice (Figure 5B). The tdT+ CMs in the large myocardial and subendocardial clusters were detected in serial transverse heart sections over distances equivalent to several CM lengths, indicating that they organized themselves along the apico-basal axis. This was confirmed by whole mount confocal sections taken from the cavity side, showing that tdT+ CMs in the endocardium region formed deep bundles in close contact with EGFP+ CMs (Figure 5F). In the myocardium, tdT+ CMs had clearly organized sarcomere structures and were of similar size as the adjacent CMs, with which they were in close contact and formed cellular junctions. Indeed, tdT+ CMs appeared structurally integrated in the myocardium and displayed normal and correctly localized intercalated discs and gap junctions with pre-existing EGFP+ CMs as revealed by N-Cadherin and Connexin-43 staining, respectively. By contrast, the tdT+ cells in the subendocardial regions were smaller and expressed lower levels of junction proteins (Figure 5G).

### 3.6. NOTCH-Independent Reduction in Cardiac Fibrosis Favors New CM Formation

**Figure 6:**
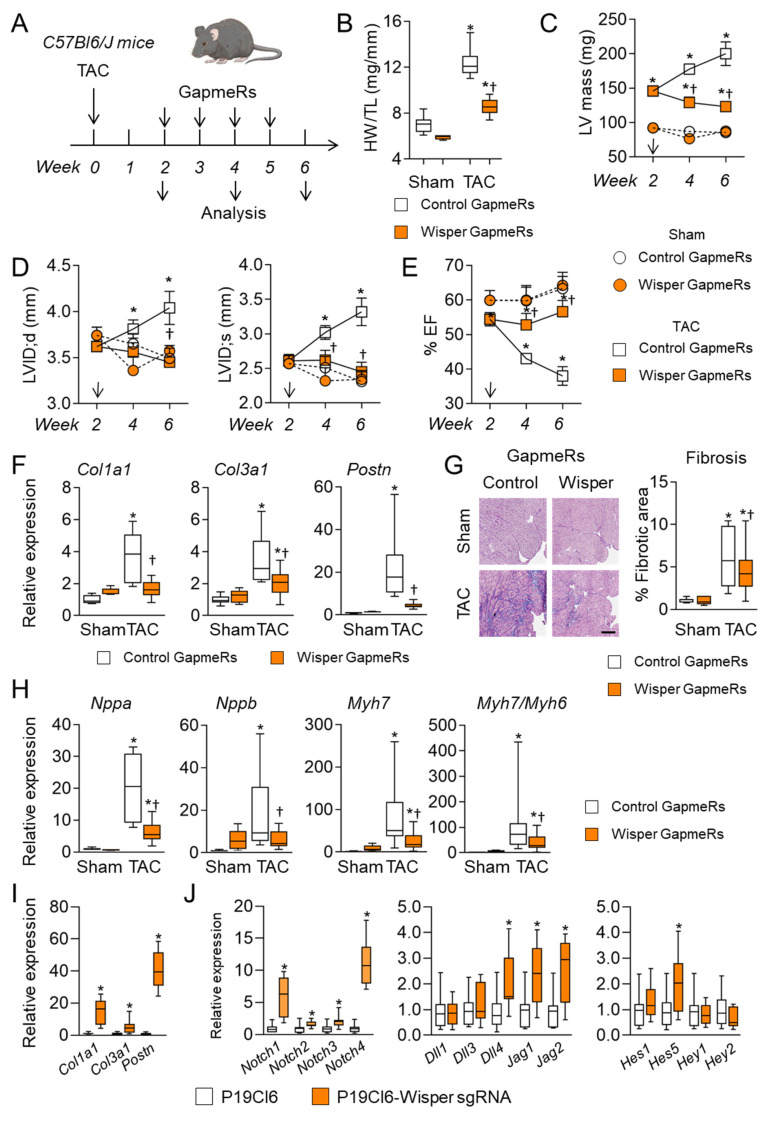
Wisper lncRNA knockdown attenuates adverse cardiac remodeling. (**A**) Experimental protocol. Mice were subjected to transaortic constriction and 2 weeks post-surgery, they received Wisper or Control GapmeRs once a week, for 4 weeks. Hearts were harvested 6 weeks after TAC. (**B**) Cardiac hypertrophy expressed as heart weight-to-tibia length ratio (mg/mm) measured at sacrifice. (**C**) Left ventricle (LV) hypertrophy (LV mass, in mg) measured by echocardiography at 2, 4 and 6 weeks post-TAC. (**D**) Remodeling of LV dimensions (in mm) in diastole and systole (LVID; d and -; s). (**E**) Functional remodeling expressed as percent ejection fraction (%EF) measured by echocardiography at 2, 4 and 6 weeks. (**F**,**G**) Wisper knockdown attenuates TAC-induced cardiac fibrosis. (**F**) Quantitative RT-PCR analysis of cardiac fibrosis markers Col1a1, Col3a1 and Postn. (**G**) Masson Trichrome staining of heart tissue sections showing interstitial fibrosis (blue) and quantification of fibrosis areas expressed as a percentage of areas of whole heart tissue sections. (**H**) RT-PCR analysis of cardiac stress markers Nppa, Nppb, Myh7 and Myh7/Myh6 ratio. In (**F**,**H**), expression level is relative to Sham-operated, Control GapmeR-treated group. *, *p* < 0.05 in TAC vs. Sham; †, *p* < 0.05 in Wisper GapmeR vs. Control GapmeR. (Sham Control-GapmeR, *n* = 6; Sham Wisper-GapmeR, *n* = 5; TAC-Control-GapmeR-4w; *n* = 6; TAC-Wisper-GapmeR-4w, *n* = 7; TAC- Control-GapmeR-6w, *n* = 9; TAC-Wisper-GapmeR-6w, *n* = 10). (**I**,**J**) Activation of Wisper expression induces fibrosis and Notch pathway genes. The P19Cl6 embryonal carcinoma cell line was transfected with a plasmid encoding dCAs9-vp16-MS2-p65-Hsf1 and a plasmid expressing guide RNA targeting Wisper promoter region to force Wisper expression. Activation of Wisper expression induces fibrosis marker genes (**I**) and Notch pathway genes (**J**), as evaluated by quantitative RT-PCR analysis. Mean ± SEM *, *p* < 0.05 in P19Cl6-Wisper sgRNA vs. P19Cl6 (*n* = 3).

Anti-NRR1 and αNRR2 treatment both promoted CM formation in the stressed heart. However, only αNRR1 antibodies efficiently restored function. Interestingly, blockade of NOTCH1 signaling was associated with a concomitant reduction in cardiac fibrosis. On the contrary, despite its favorable effect on CM production, the αNRR2 treatment was unable to reduce fibrosis and remodeling. The fibrotic component appeared therefore as a clear determinant of heart dysfunction in this model. Hence, we decided to evaluate whether a simple anti-fibrotic treatment could create in itself favorable conditions for CM production. The lncRNA *Wisper* has been demonstrated to be a crucial regulator of cardiac fibrosis [15]. Cardiac *Wisper* expression was induced upon pressure overload. More importantly, its expression was decreased after αNRR1 but not αNRR2 treatment (Figure 2A). *Wisper* can be targeted by anti-*Wisper* GapmeRs to prevent the development of fibrosis in the infarcted heart and promote functional recovery [15]. To determine whether *Wisper* knockdown could also induce regression of established cardiac remodeling in a pressure overload model, mice received *Wisper* GapmeRs at 2, 3, 4 and 5 weeks after TAC (Figure 6A). Two weeks post-surgery, TAC induced severe cardiac remodeling as shown by increased heart weight-to-tibia length ratio and left ventricular (LV) mass (Figure 6B,C). These parameters deteriorated further in animals receiving a control GapmeR during the following weeks. Cardiac dilation developed and, as a consequence, cardiac function declined (Figure 6D,E). In sharp contrast, *Wisper* silencing protected the heart from adverse cardiac remodeling. At 6 weeks, *Wisper* GapmeR-treated mice were characterized by normal cardiac dimensions and function (Figure 6B–E and Supplementary Table S3). As expected, the expression of cardiac fibrosis marker genes (*Col1a1*, *Col3a1*, *Postn*) as well as collagen deposition were decreased after *Wisper* GapmeR administration (Figure 6F,G). In addition, the induction of cardiac stress marker genes *Myh7*, *Nppa* and *Nppb* was also blunted, consistent with improved function following *Wisper* knockdown (Figure 6H).

We next determined whether *Wisper* silencing affected the activity of the NOTCH pathway. For this, we investigated expression of genes encoding components of the NOTCH pathway following *Wisper* knockdown in sham and TAC operated animals. Globally, we observed a general downregulation of NOTCH family members and target genes in *Wisper* GapmeR-treated mice (Supplementary Figure S5A). To examine whether NOTCH genes could be under control of *Wisper*, we used a dCas9-based synergistic activation mediator (SAM) system [36] to force the expression of *Wisper* in P19Cl6 cells. As previously described [15], induction of *Wisper* resulted in the expression of fibrosis marker genes (*Col1a1*, *Col3a1*, *Postn*) in this normally non-fibroblastic cell line, demonstrating the efficacy of the manipulation (Figure 6I and Supplementary Figure S5B). More importantly, the expression of several members of the NOTCH pathway, specifically *Notch1*, *Notch4*, *Jagged1*, *Jagged2*, and *Hes5* were significantly induced upon *Wisper* activation (Figure 6J). Altogether, this series of experiments suggested the existence of crosstalk between the NOTCH pathway and a crucial regulator of fibrosis, which could determine outcome in the diseased heart.

**Figure 7:**
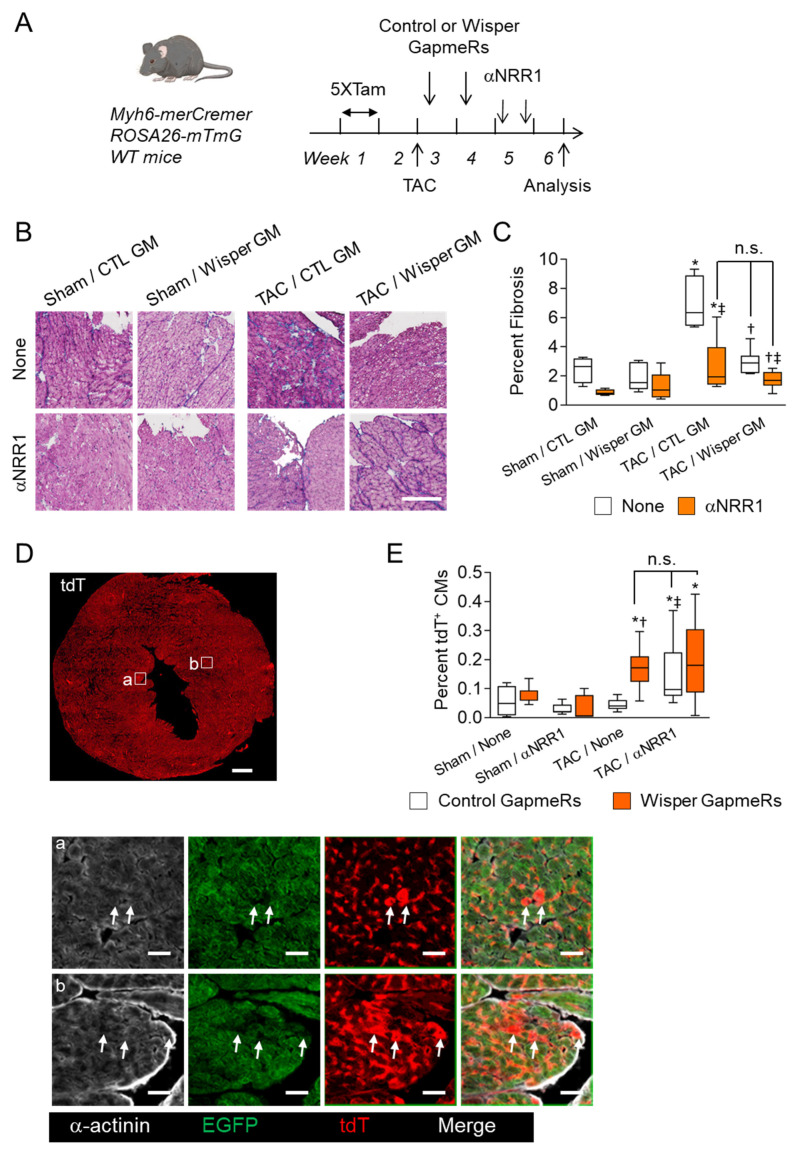
Concerted expression and action of Wisper and NOTCH1 signaling on cardiac fibrosis and new myocyte formation. (**A**) Experimental protocol. Tamoxifen-treated (5 consecutive days) Myh6-MerCreMer;ROSA26-mTmG reporter mice were subjected to transaortic constriction or Sham operation and 2 and 9 days post-surgery, they received Wisper-GapmeR or Control (Cont.)-GapmeR. Two weeks after TAC, they were treated twice with αNRR1 antibody at 4 days interval or left untreated (None). The hearts were harvested 4 weeks after TAC and analyzed. (**B**) Masson Trichrome of heart tissue sections showing collagen deposition (blue). (**C**) Quantitative analysis of cardiac fibrosis expressed as percent of whole-heart sectional area. (**D**,**E**) Wisper knockdown stimulates formation of new CMs. (**D**) Fluorescence photomicrograph of a whole heart section showing tdT+ CMs in the endocardium and myocardium regions. The areas within squares are displayed at higher magnifications demonstrating the presence of α-actinin+ (gray), tdT+ CMs (red) in the endocardium (**a**) and myocardium (**b**) in contact with pre-existing EGFP+ CMs (green). (**E**) Quantification of new tdT+ CM formation expressed as percent of total number of CMs per whole heart tissue section. The graphs in (**E**) show mean ± SEM. *, *p* < 0.05 in TAC vs. Sham; †, *p* < 0.05 in Wisper-GapmeR vs. Control-GapmeR; ‡, *p* < 0.05 in αNRR1 vs. None and n.s., not significant (Sham Control-GapmeR αNRR1, *n* = 3; Sham Wisper-GapmeR αNRR1, *n* = 3; TAC Control-GapmeR αNRR1, *n* = 5; TAC Control-GapmeR None, *n* = 3; TAC-Control-GapmeR αNRR1, *n* = 6; TAC Wisper-GapmeR None, *n* = 6; TAC Wisper-GapmeR αNRR1, *n* = 7). Scale bars in (**B**) = 100 μm; in (**D**) = 1 mm, insets = 50 μm.

Because Wisper-mediated pathways and NOTCH signaling appeared to be coordinately regulated, we sought to determine whether Wisper silencing could also induce new CM formation. Therefore, Myh6-MerCreMer; ROSA26-mTmG mice were subjected to TAC. At days 2 and 9 days post-surgery, groups of mice were administered with Wisper GapmeRs. Other groups were treated at days 15 and 19 after surgery with two injections of αNRR1 antibody. Finally, some mice received both treatments to evaluate potential synergistic effects of Wisper silencing and NOTCH1 pathway inhibition (Figure 7A). After 4 weeks of TAC, mice that received control GapmeRs developed overt fibrosis, which was significantly blunted by Wisper GapmeRs or αNRR1 treatments, confirming their respective anti-fibrotic effects (Figure 7B,C). Combined αNRR1 and Wisper GapmeR treatments further decreased fibrosis to levels observed in sham-operated mice. Furthermore, lineage tracing analysis showed that newly formed tdT+ CMs were found in the myocardium and the subendocardial region, integrated with pre-existing EGFP+ CMs (Figure 7D,E). In TAC mice, Wisper GapmeR treatment induced a significant increase in tdT+ CMs relative to mice treated with a control GapmeR, comparable to what is observed in mice treated with αNRR1 antibody. Combined αNRR1 and Wisper GapmeR treatment appeared to further increase CM production, although not significantly.

## 4. Discussion

Prolonged pressure overload in the left ventricle leads to cardiac hypertrophy, cardiac fibrosis and deterioration of cardiac function. Here, we show that sequential activation and inhibition of the NOTCH1 receptor, but not of the NOTCH2 receptor, prevents cardiac fibrosis, remodeling and loss of cardiac function as assessed 4 weeks after transaortic constriction (please see Graphical Abstract). Interestingly, both NOTCH1 and NOTCH2 blockade, if associated with prior activation, promote new CM formation from non-myocyte origin. However, the main difference between the two treatments consists in the inefficacy of NOTCH2 inhibition to reduce fibrosis and subsequent remodeling. NOTCH2 blockade resulted in a significant increase in NOTCH receptor expression. In particular, NOTCH1 is induced following αNRR2 administration (Supplementary Figure S2). Considering that NOTCH1 blockade produces beneficial effects on fibrosis, this compensatory response via NOTCH1 expression in mice treated with αNRR2 likely contributes to fibrosis development and deterioration of function. Knockdown of the cardiac fibroblast-enriched lncRNA *Wisper* exerts similar beneficial effects as NOTCH1 activation and inhibition on cardiac fibrosis and remodeling, and therefore on cardiac function, during the response to pressure overload. Furthermore, *Wisper* silencing also favors the emergence of new CMs. It is important to note that the beneficial effects of *Wisper* knockdown cannot be due to a direct action on cardiomyocytes, as these cells have no detectable expression of this cardiac fibroblast-enriched lncRNA [15]. Nevertheless, transdifferentiation is a rare event, and recovery of function cannot be explained by the sole production of cardiomyocytes from non-myocyte origin. Maladaptive cardiac fibrosis is central to the development of heart failure and the therapeutic potential of targeting cardiac fibrosis to prevent cardiac dysfunction is well-recognized [15,37,38]. In this regard, it is noteworthy that *Wisper* knockdown both prevented and reversed established cardiac fibrosis under pressure overload. Since all three treatments favor CM formation but only NOTCH1 blockade and *Wisper* knockdown preserve function, it is likely that function is sustained secondary to decreased fibrosis. Interestingly, *Wisper* silencing resulted in an overall downregulation of the NOTCH1 signaling pathway, mimicking thereby the situation observed following NOTCH1 blockade. On the contrary, NOTCH2 inhibition induced *Notch1* and other NOTCH receptor expression. The level of NOTCH1 activity appears therefore to play an important role in determining outcome.

NOTCH1 signaling has been implicated in fibrosis in several ways [39]. First, the NOTCH pathway protects CMs in the damaged heart via limiting hypertrophy. Specifically, NOTCH activation is beneficial in the CM population by enhancing myocyte survival. In doing so, NOTCH contributes indirectly to reducing the compensatory fibrotic response. We have indeed shown that JAGGED1-mediated NOTCH signaling in CMs attenuates cardiac hypertrophy and reduces fibrosis after TAC [28]. Administration of a JAGGED1-containing hydrogel in the infarcted heart also reduces cardiac fibrosis and improves function [40]. Accordingly, deletion of the NOTCH ligand DELTA-LIKE1 promotes myofibroblast differentiation [41]. Downregulation of NOTCH signaling also appears necessary for TGFβ-induced fibroblast-to-myofibroblast transformation in the stressed heart [42]. Importantly, this biological process is mediated by NOTCH1 but not NOTCH2. On the other hand, in different organ systems, and in accordance with what was seen in the present study, inhibition of NOTCH signaling has been shown to prevent the development of fibrosis following injury [43,44,45]. In this context, we used a model of pressure overload, which is characterized by a limited inflammatory response as compared to, for instance, myocardial infarction. Then, our lineage tracing experiments shows that the progeny of NOTCH-activated cells are in vast majority endothelial cells. Interestingly, JAGGED1 and NOTCH1 have been directly involved in controlling endothelial-to-myofibroblast transition (EndoMT), and then organ fibrosis [46]. EndoMT is essential during development of the heart, a process that is controlled in part by the NOTCH pathway, and has been implicated in the development of myocardial fibrosis [47]. Interestingly, EndoMT is a dynamic and reversible process, resulting in so-called partial EndoMT, in which endothelial cells express mesenchymal markers without fully committing to the fibroblastic lineage. In the same vein, previous work demonstrated that epicardial cells and endothelial cells are targets of NOTCH action in the injured heart and contribute to fibrotic repair [27]. Epicardium-derived cells are known to give rise to both an endothelial and fibroblastic progeny. Indeed, NOTCH activates a common precursor between the two lineages [27]. Thus, NOTCH signaling occurs in particular in NMCs capable of a certain degree of plasticity, which depends on the environmental conditions experienced at the time of activation.

Cardiac NOTCH signaling also allows the emergence of CPCs in the stressed heart [28]. Nevertheless, when chronically activated, the NOTCH pathway interferes with CM terminal differentiation. Evidence indeed suggests that downregulation of NOTCH signaling is a prerequisite for CM production [24]. Here, we show that NOTCH1 and NOTCH2 blockade increase the number of BrdU+ CMs in response to pressure overload. Importantly, our analysis of CM nucleation shows that BrdU+ CMs were largely mono-nucleated, a feature compatible with newly formed CMs. Because NOTCH activation promotes the development of CPCs in the injured heart, we hypothesized that BrdU+ CMs could derive from proliferating CPCs undergoing differentiation upon NOTCH blockade. This assumption was also supported by our work in human primary CPCs [48]. Indeed, sequential activation and inhibition of the NOTCH pathway promotes commitment into the cardiogenic lineage and CM differentiation of human adult atrial precursors from mesenchymal origin. Herein, we formally demonstrate using lineage tracing that NOTCH manipulation in NMCs stimulates their transdifferentiation into CMs. Nevertheless, the nature of the precursors is not completely established. An important subset of the small tdT+ CMs is located in the subendocardium. Interestingly, NOTCH1-traced cells are abundantly present in the endocardium of the heart subjected to hemodynamic overload. During cardiac development, the endocardium controls cardiac chamber formation through NOTCH1 signaling [49,50], suggesting that some dormant developmental mechanisms might be reactivated under our experimental conditions. Of note, highly plastic endothelial cells from endocardial and vascular origin have been implicated in the formation of reparative structures in the damaged heart [51]. In addition, a subset of cardiac fibroblasts have been shown to express a CM gene signature [52] and suggested to be primed for direct transdifferentiation into myocytes [53]. Along this line, fibroblast reprogramming into induced CPCs is enhanced by concomitant NOTCH inhibition [29]. Therefore, the emergence of tdT+ CMs under NOTCH blockade may also result from the recruitment of fibroblasts into the cardiogenic lineage. Nonetheless, these remain rare events, which cannot contribute by themselves to the recovery of function. Detection of NMC-derived CMs should be seen therefore as a signature of NOTCH action in the heart as a modulator of both cardiogenesis and fibrosis.

Fibrotic and regenerative repairs are thought to be mutually exclusive. In the present study, we demonstrate that the NOTCH1 signaling pathway regulates both the development of fibrosis and the emergence of CMs from non-myocyte origin. However, whether these two processes are coordinately regulated is still unclear. By limiting the extent of cardiac fibrosis, NOTCH1 activity in the heart creates a permissive environment for new CM production. In this regard, *Wisper* knockdown is also associated with increased CM formation in the stressed heart. *Wisper* is a cardiac fibroblast identity lncRNA that controls the development of fibrosis [15]. Therefore, similar to NOTCH inhibition, *Wisper* silencing might facilitate cardiogenesis in the heart by alleviating the detrimental effects resulting from the hostile fibrotic environment. On the other hand, *Wisper* downregulation could favor transdifferentiation by enabling the expression of a cardiogenic gene program that is normally repressed in fibroblasts under conditions of stress such as that encountered in the heart subjected to pressure overload. In a similar vein, NOTCH modulation in NMCs could promote CM production by stimulating a cardiac fate via repressing fibroblastic specification. Altogether, our study demonstrates therefore that cardiogenesis can be stimulated in the adult mammalian heart by limiting cardiac fibrosis, and pave the way for future work aimed at promoting regenerative repair in the damaged myocardium.

## Data Availability

Data will be provided by the authors upon reasonable request.

## Supplementary Materials

The following supporting information can be downloaded at: https://www.mdpi.com/article/10.3390/jcdd9040111/s1, Table S1: List of primers, GapmeRs, TaqMan probes and antibodies; Table S2: Echographic parameters in TAC mice administered with either anti-NRR1 or anti NRR2 antibodies; Table S3: Echographic parameters in TAC mice administered with anti-*Wisper* GapmeRs; Figure S1: The NOTCH pathway is activated in non-myocyte cells under pressure overload; Figure S2: Upregulation of NOTCH receptor expression in anti-NRR2 treated mice; Figure S3: Anti-NRR1 treatment blocks NOTCH1 signaling in the heart; Figure S4: Conversion from tdT- to EGFP-expressing cardiomyocytes in MerCreMer; ROSA26-mTmG double transgenic mice upon tamoxifen administration; Figure S5: *Wisper* knockdown is associated with downregulation of the NOTCH pathway in the heart.
