# Supplementary material for: Inhibition of the NOTCH1 Pathway in the Stressed Heart Limits Fibrosis and Promotes Recruitment of Non-Myocyte Cells into the Cardiomyocyte Fate
Source: J Cardiovasc Dev Dis. 2022 Apr 7;9(4):111. doi: 10.3390/jcdd9040111 (PMC9024539; doi:10.3390/jcdd9040111)
Supplement: Supplementary file 1 [file jcdd-09-00111-s001.zip › jcdd-1573057-supplementary.pdf]

Inhibition of the NOTCH1 pathway in the stressed heart limits fibrosis and promotes recruitment of non-myocyte cells into the cardiomyocyte fate

Mohamed Nemir, Maryam Kay, Damien Maison, Corinne Berthonneche, Alexandre Sarre, Isabelle Plaisance, Thierry Pedrazzini

Online Supplementary Material

List of Supplementary Tables and Figures

Suppl. Table S1	List of primers, GapmeRs, TaqMan probes and antibodies.
Suppl. Table S2	Echographic parameters in TAC mice administered with either anti-NRR1 or anti NRR2 antibodies.
Suppl. Table S3	Echographic parameters in TAC mice administered with anti-Wisper GapmeRs.
Suppl. Figure S1	The NOTCH pathway is activated in non-myocyte cells under pressure overload.
Suppl. Figure S2	Upregulation of NOTCH receptor expression in anti-NRR2 treated mice.
Suppl. Figure S3	Anti-NRR1 treatment blocks NOTCH1 signaling in the heart.
Suppl. Figure S4	Conversion from tdT- to EGFP-expressing cardiomyocytes in MerCreMer; ROSA26-mTmG double transgenic mice upon tamoxifen administration.
Suppl. Figure S5	Wisper knockdown is associated with downregulation of the NOTCH pathway in the heart.

Supplementary Table S1: List of primers, GapmeRs, TaqMan probes and antibodies.

Target	Sequence/ Assay	Source	
Wisper Left Primer	CTGCTTCTCCAAAAGCCAAG	microsynth.ch	
Wisper Right Primer	TAGACGAGCTGCTTCCCAGT	microsynth.ch	
Gapdh Left Primer	TGCACCACCAACTGCTTAGC	microsynth.ch	
Gapdh Right Primer	GGCATGGACTGTGGTCATGAG	microsynth.ch	
Control GapmeR	AGGTGTGCGATAGAG	Exiqon (qiagen.com)	
Wisper GapmeR	AACACGTCTATACGC	Exiqon (qiagen.com)	
Myh6	Mm00440354_m1	Applied Biosystems	
Myh7	Mm00600555_m1	Applied Biosystems	
Nppa	Mm01255747_g1	Applied Biosystems	
Nppb	Mm01255770_g1	Applied Biosystems	
Acta1	Mm00808218_g1	Applied Biosystems	
Ctgf	Mm01192933_g1	Applied Biosystems	
TGFb2	Mm00436955_m1	Applied Biosystems	
Col1a1	Mm00801666_g1	Applied Biosystems	
Col3a1	Mm00802331_m1	Applied Biosystems	
Postn	Mm00450111_m1	Applied Biosystems	
Notch1	Mm00435245_m1	Applied Biosystems	
Notch2	Mm00803077_m1	Applied Biosystems	
Notch3	Mm00435270_m1	Applied Biosystems	
Notch4	Mm00440525_m1	Applied Biosystems	
Dll1	Mm00432841_m1	Applied Biosystems	
Dll3	Mm00432854_m1	Applied Biosystems	
Dll4	Mm00444619_m1	Applied Biosystems	
Jag1	Mm00496902_m1	Applied Biosystems	
Jag2	Mm00439935_m1	Applied Biosystems	
Hes1	Mm00468601_m1	Applied Biosystems	
Hes5	Mm00439311_g1	Applied Biosystems	
Hey1	Mm00468865_m1	Applied Biosystems	
Hey2	Mm00469280_m1	Applied Biosystems	
Antibody	Source and Cat. #	RRID	Dilution
Mouse anti-a-actinin	Sigma-Aldrich Cat# A7811	AB_476766	1:500
Mouse anti-a-actinin	Alexa-Fluor 647 / 488-conjugated	in house	1:250
Rabbit anti-laminin	Sigma-Aldrich Cat# L9393	AB_477163	1:500
Rabbit anti-laminin	Alexa-Fluor 647 / 488-conjugated	in house	1:250
Rabbit anti-GATA4	Abcam Cat# ab134057	AB_2725747	1:250
Rat anti-BrdU	Abcam Cat# ab6326	AB_305426	1:400
Rat anti-Sca-1	Abcam Cat# ab51317	AB_1640946	1:1000
Rabbit anti-Periostin	Abcam Cat# ab14041	AB_2299859	1:500
Rabbit anti-N-Cadheri	Abcam Cat# ab12221	AB_298943	1:100
Rabbit anti-PDGFRa	Abcam Cat# ab61219	AB_2162341	1:100
Rabbit anti-Connexin-	Sigma-Aldrich Cat# C6219	AB_476857)	1:500
Goat anti-vimentin	Sigma-Aldrich Cat# V4630	AB_477619	1:150
Rat anti-CD31	Thermo Fisher Scientific Cat# 14-0311-8	AB_467201	1:300
Chicken anti-EGFP	Abcam Cat# ab13970	AB_300798	1:1000

Supplementary Table S2: Echographic parameters in TAC mice administered with either anti-NRR1 or anti NRR2 antibodies.

Group	BW (g)	LVM (mg)	LVM/BW (mg/g)	LVID;d (mm)	LVID;s (mm)	LVPW;d (mm)	LVPW;s (mm)	%EF	AoV (mm/s)	n =
WT-Sham-None	44.7 ± 1.4	136.5 ± 6.3	3.1 ± 0.1	4.1 ± 0.1	2.5 ± 0.1	0.9 ± 0	1.3 ± 0.1	69.9 ± 1.9	962.5 ± 57.2	7
WT-TAC-None	43.8 ± 1.4	232.5 ± 19.4 *	5.3 ± 0.5 *	4.2 ± 0.2	3.1 ± 0.2 *	1.2 ± 0 *	1.5 ± 0 *	54.3 ± 4.6*	3267.4 ± 224.1 *	8
TgJ1-Sham-None	36.3 ± 2.5	83.7 ± 7.1 ‡	2.3 ± 0.2 ‡	3.4 ± 0.2 ‡	2.1 ± 0.2‡	0.8 ± 0 ‡	1.1 ± 0 ‡	68 ± 2.7	732.2 ± 39.3	7
TgJ1-TAC-None	39.8 ± 2.4	152.9 ± 20.7 *‡	3.8 ± 0.4*‡	3.7 ± 0.2 ‡	2.5 ± 0.3	1 ± 0.1 *‡	1.4 ± 0.1 *	62.1 ± 5.6	3259.3 ± 269.5 *	7
WT-Sham-αNRR1	45 ± 1.8	122.7 ± 9.2	2.7 ± 0.2	3.9 ± 0.1 †	2.5 ± 0.1	0.9 ± 0	1.2 ± 0.1	65.8 ± 1.9	837 ± 77.2	6
WT-TAC-αNRR1	41.4 ± 1.7	180.5 ± 13.2 *†	4.3 ± 0.2*†	4 ± 0.1	2.6 ± 0.2	1.1 ± 0.1*†	1.5 ± 0.1 *	63.8 ± 3.8	4112.9 ± 213.5 *†	9
TgJ1-Sham-αNRR1	38 ± 1.2	78.3 ± 7.9 ‡	2.1 ± 0.2	3.1 ± 0.2 ‡	1.8 ± 0.1‡	0.8 ± 0 ‡	1.1 ± 0.1 ‡	75.6 ± 2.9†	753.4 ± 33.6	7
TgJ1-TAC-αNRR1	39.4 ± 1.3	109.2 ± 13.1*†‡	2.8 ± 0.4*‡	3.1 ± 0.2 ‡	1.8 ± 0.2 †	1 ± 0 *†‡	1.4 ± 0.1 *	76.1 ± 4 †‡	4069.4 ± 289.4 *†	7
WT-Sham-αNRR2	39.8 ± 7.1	99.1 ± 13.8 †	2.6 ± 0.3	4.2 ± 0.2	2.8 ± 0.3	0.7 ± 0.1 †	1.1 ± 0.1 †	61.8 ± 6.3	972 ± 84.1	5
WT-TAC-αNRR2	44.8 ± 2.2	219.5 ± 14.9 *	5 ± 0.4 *	4.8 ± 0.2*†	3.6 ± 0.2 *	1.1 ± 0 *†	1.4 ± 0.1 *	51.4 ± 4 *	3492.1 ± 241.9 *	9
TgJ1-Sham-αNRR2	38 ± 2.1	87.4 ± 9.4	2.3 ± 0.2	3.8 ± 0.2	2.4 ± 0.2‡	0.8 ± 0.1	1.4 ± 0.1 †‡	69.5 ± 2.3	1103.1 ± 144.6 †	5
TgJ1-TAC-αNRR2	44.8 ± 1.4	149 ± 11.7 *‡	3.4 ± 0.3*‡	3.9 ± 0.2 ‡	2.6 ± 0.2	1.1 ± 0.1 *	1.5 ± 0.1	65.5 ± 4.4‡	3306.1 ± 162.9 *	10

WT and TgJ1 mice were subjected to aortic constriction (TAC) or to Sham operation (Sham), as in Figure 1A. Two weeks later, the mice received either no antibody (None), NOTCH1 (αNRR1) or NOTCH2 (αNRR2) blocking antibodies. Echocardiography measurements were performed 4 weeks post-surgery. Abbreviations are: BW, body weight, LVM, LV mass; LVM/BW, LV mass-to-body weight ratio; LVID;d, left ventricle internal diameter in diastole;-s, in systole; LVPW;d, left ventricle posterior wall in diastole; -s, in systole; %EF, percent ejection fraction; AoV, aortic velocity. The values represent mean ± SEM. *, $p<0.05$ in TAC vs. Sham; †, in NRR1/2 vs. None; ‡, in TgJ1 vs. WT. The number of animals per group is indicated in the right-most column (n =).

Supplementary Table S3: Echographic parameters in TAC mice administered with anti-Wisper GapmeRs.

Group	BW (g)	LVM (mg)	LVM/BW (mg/g)	LVID;d (mm)	LVID;s (mm)	LVPW;d (mm)	LVPW;s (mm)	%EF	AoV (mm/s)	n =
Sham Control-GapmeR	27.8 ± 0.7	85.6 ± 4.2	3.1 ± 0.2	3.5 ± 0.1	2.3 ± 0.1	0.8 ± 0	1.0 ± 0	63.3 ± 3.6	817.2 ± 39.6	6
TAC Control-GapmeR	27.7 ± 0.2	200.1 ± 17.3 *	7.2 ± 0.7 *	4.0 ± 0.2 *	3.3 ± 0.2 *	1.2 ± 0.0 *	1.4 ± 0.0 *	38.0 ± 2.7 *	4783.0 ± 52.6 *	9
Sham Wisper-GapmeR	26.4 ± 0.5	87.4 ± 3.9	3.3 ± 0.2	3.6 ± 0.1	2.3 ± 0.1	0.7 ± 0	1.0 ± 0	64.2 ± 3.8	892.9 ± 42.5	5
TAC Wisper-GapmeR	25.9 ± 0.5	123.0 ± 4.8 *†	4.8 ± 0.2 *†	3.5 ± 0.1 †	2.5 ± 0.1 †	1.0 ± 0.0 *†	1.3 ± 0.0 *	56.6 ± 3.2 †	4512.5 ± 201.7 *	10

Mice were subjected to aortic constriction (TAC) or to Sham operation (Sham), as in Figure 6A. Two weeks later, the mice received weekly injections of either Control-GapmeR or Wisper-GapmeR, for 4 consecutive weeks. The table presents echocardiography measurements performed at 6 weeks post-surgery. Abbreviations are: BW, body weight, LVM, LV mass; LVM/BW, LV mass-to-body weight ratio; LVID;d, left ventricle internal diameter in diastole;-s, in systole; LVPW;d, left ventricle posterior wall in diastole; -s, in systole; %EF, percent ejection fraction; AoV, aortic velocity. The values represent mean ± SEM. *, $p<0.05$ in TAC vs. Sham; †, Wisper-GapmeR vs. Control-GapmeR. The number of animals per group is indicated in the right-most column (n =).

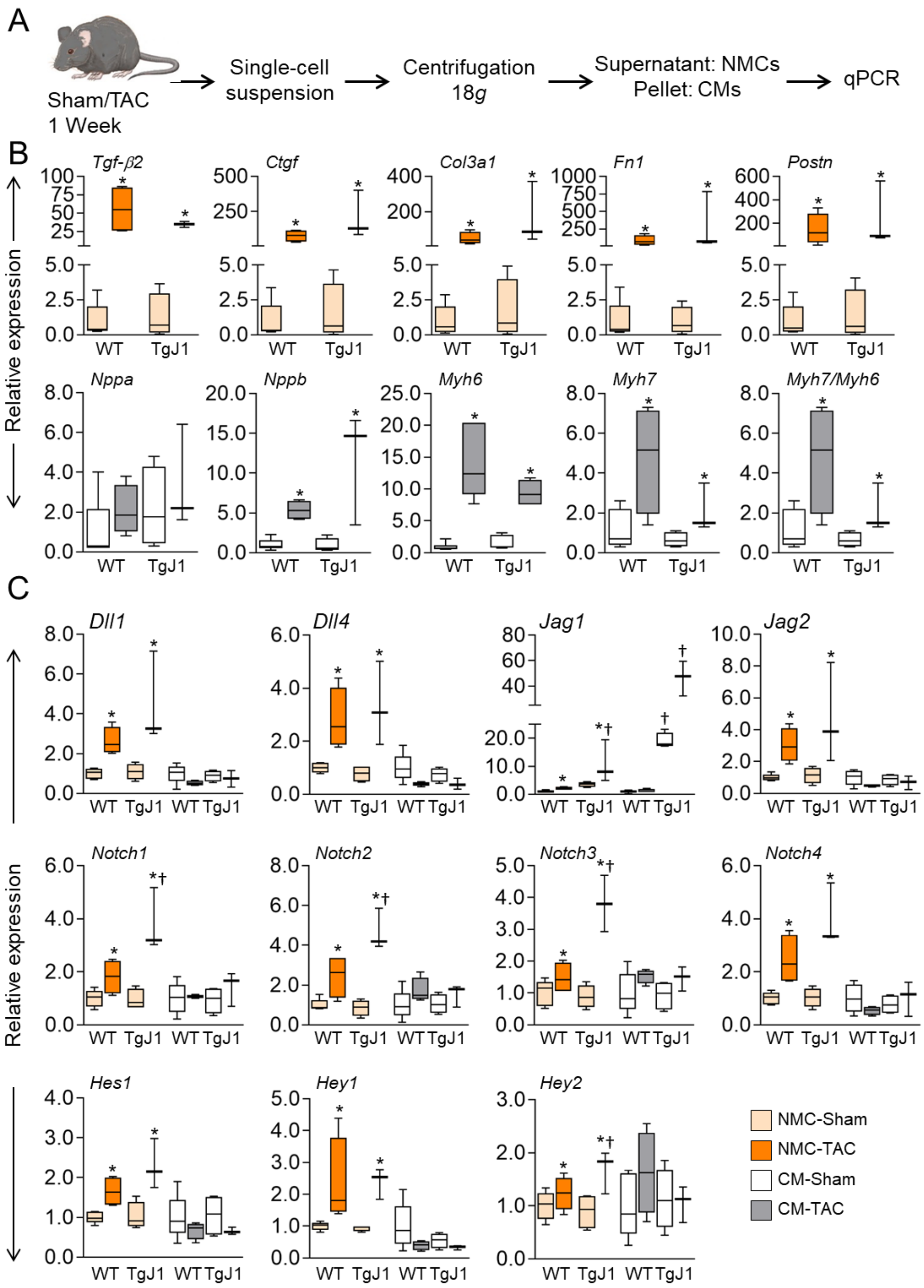

Supplementary Figure S1. The NOTCH pathway is activated in non-myocyte cells under pressure overload. (**A**). WT and TgJ1 mice were subjected to Sham or TAC surgery. After 1 week, the hearts were harvested and the non-myocyte (NMCs) cells and cardiomyocytes (CMs) were isolated by enzymatic digestion and differential centrifugation and processed for total cellular RNA isolation and quantitative RT-PCR analyses. (**B**). Expression level of fibrosis marker genes (Tgf- 2,Ctgf, Col3a1, Fn1 and Postn), and cardiomyocyte stress genes (Nppa, Nppb, Myh6, Myh7 and Myh7/Myh6 ratio). (**C**) Expression level of NOTCH receptors (Notch1-Notch4), ligands (Dll1, Dll4, Jag1 and Jag2) and target genes (Hes1, Hey1 and Hey2). The expression levels are relative to Sham-operated WT mice (WT Sham, n = 5; WT TAC, n = 4; TGJ1 Sham, n = 4; TGJ1 TAC, n = 3). *, p<0.05 in TAC vs. Sham; †, p<0.05 in TGJ1 vs. WT.

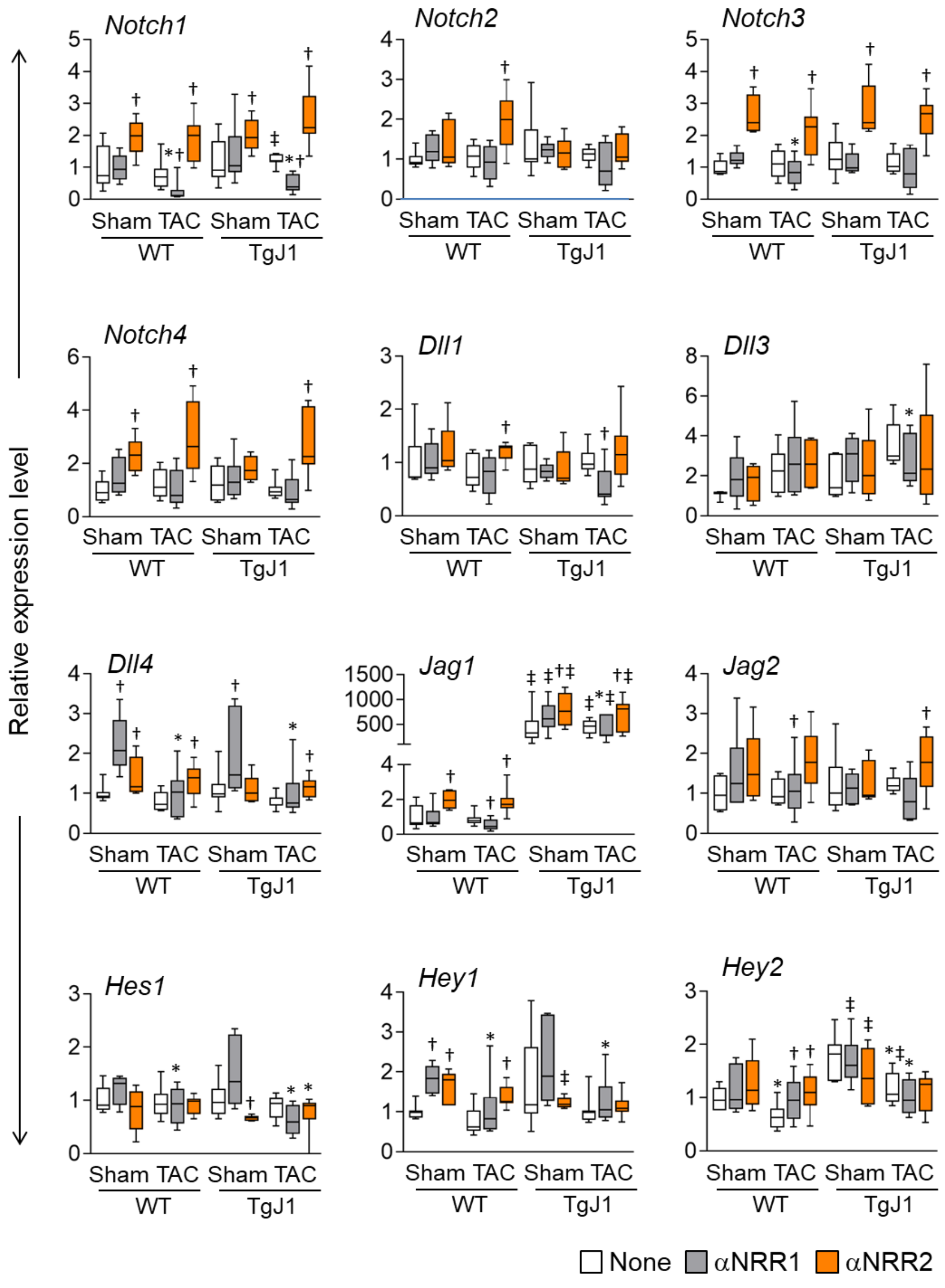

Supplementary Figure S2. Upregulation of NOTCH receptor expression in anti-NRR2 treated mice. WT and TGJ1 mice were subjected to TAC or Sham operation, as in Figure 1A. After 2 weeks, they were injected with αNRR1 or αNRR2. AT 4 weeks post-surgery, the hearts were harvested and subjected to quantitative RT-PCR analysis of gene expression of NOTCH receptors (Notch1-Notch4), ligands (Dll1, Dll2, Dll4, Jag1 and Jag2) and target genes (Hes1, Hey1 and Hey2). The levels of expression are relative to Sham-operated, untreated WT mice. The number of mice per group is indicated in Supplementary Table 1. *, p<0.05 in TAC vs. Sham; †, p<0.05 in NRR vs. None; ‡, p<0.05 in TGJ1 vs. WT.

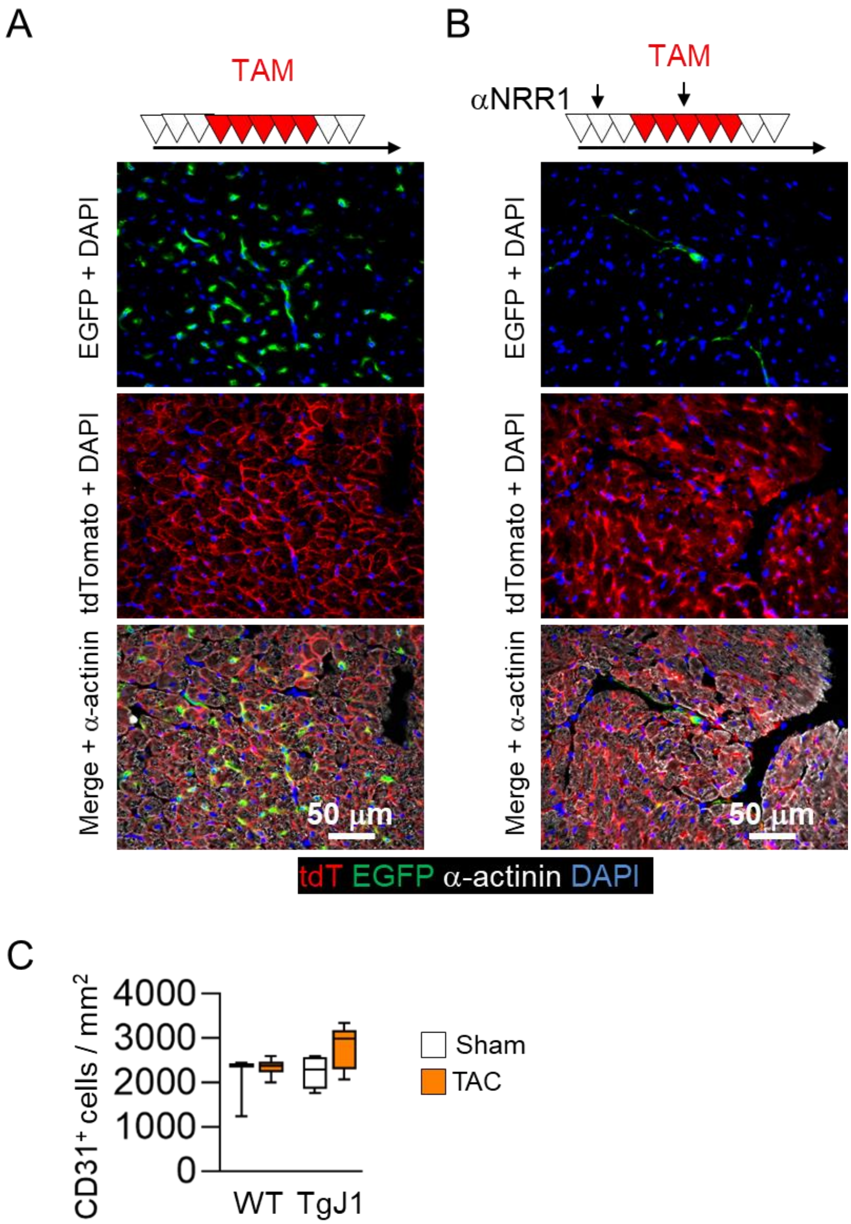

Supplementary Figure S3. Anti-NRR1 treatment blocks NOTCH1 signaling in the heart. (**A**) N1IP-CreErt2;ROSA26-mTmG NOTCH1 reporter mice were injected with Tamoxifen to induce conversion from tdT to EGFP expression in cells experiencing NOTCH1 signaling. (**B**) Mice were injected with anti-NRR1 antibody to block NOTCH1 signaling prior to and during Tamoxifen administration. Two days after the last Tamoxifen injection, the hearts were harvested and processed for immunostaining to reveal NOTCH1-traced EGFP-positive cells (Green), tdT-positive non-traced cells (Red) and α-actinin (Gray). Note the reduced number of EGFP+ cells in presence of anti-NRR1 injection. Scale bar = 50 μm. (**C**). Capillary density ($CD31^+$ cells / mm^2) in WT and TGJ1 mice subjected to Sam and TAC surgery and αNRR1 treatment, as described in Figure 4.

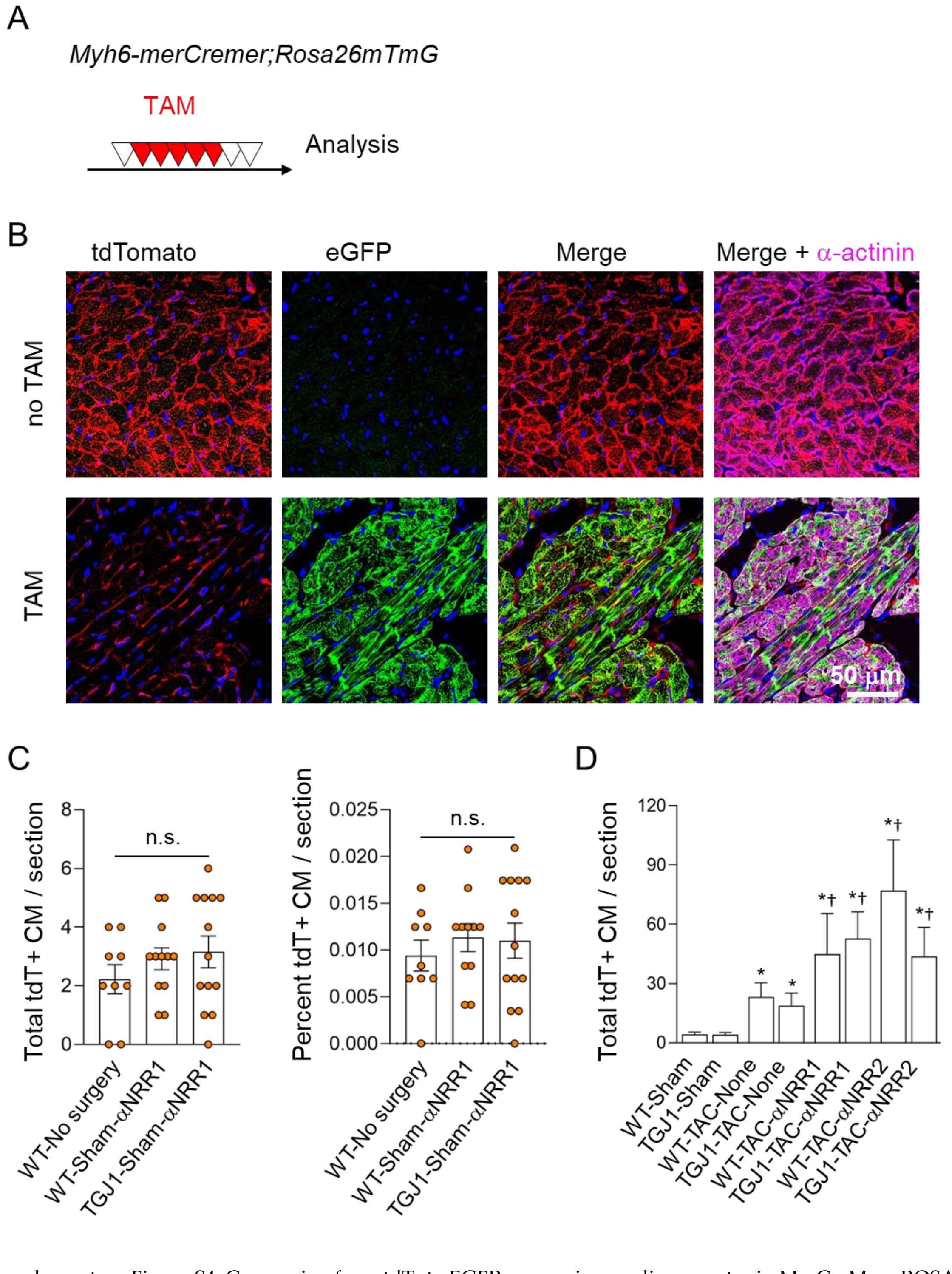

Supplementary Figure S4. Conversion from tdT- to EGFP-expressing cardiomyocytes in MerCreMer ; ROSA26-mTmG double transgenic mice upon tamoxifen administration. (**A**). Myh6-MerCreMer;ROSA26-mTmG mice received 5 daily injections of Tamoxifen to induced recombination of the ROSA26-mTmG locus. (**B**). The hearts were harvested two days after the last Tamoxifen injection and processed for immunostaining to reveal α-actinin-positive (Gray) converted EGFP-positive CMs (Green) and tdT-positive non-converted NMCs (Red). Note the nearly complete conversion of CMs from tdT to EGFP expression, and the lack of EGFP expression in NMCs, which remained tdT-positive. Scale bar = 50 µm. (**C**). Number and percentage of tdT+ cardiomyocytes per transverse heart tissue section in the untouched WT heart and in sham-operated WT and TGJ1 hearts following Tamoxifen-induced conversion as an indication of the amounts of CMs escaping conversion into GFP-expressing CMs. (**D**). Quantification of new tdT+ CMs in WT and TGJ1 mice subjected to Sham or TAC operation and treated with αNRR1, αNRR2 antibodies or none. The data show numbers of tdT+ CMs per heart section *, $p<0.05$ in TAC vs. Sham; †, $p<0.05$ in αNRR vs. None (n = 4-8 mice per group).

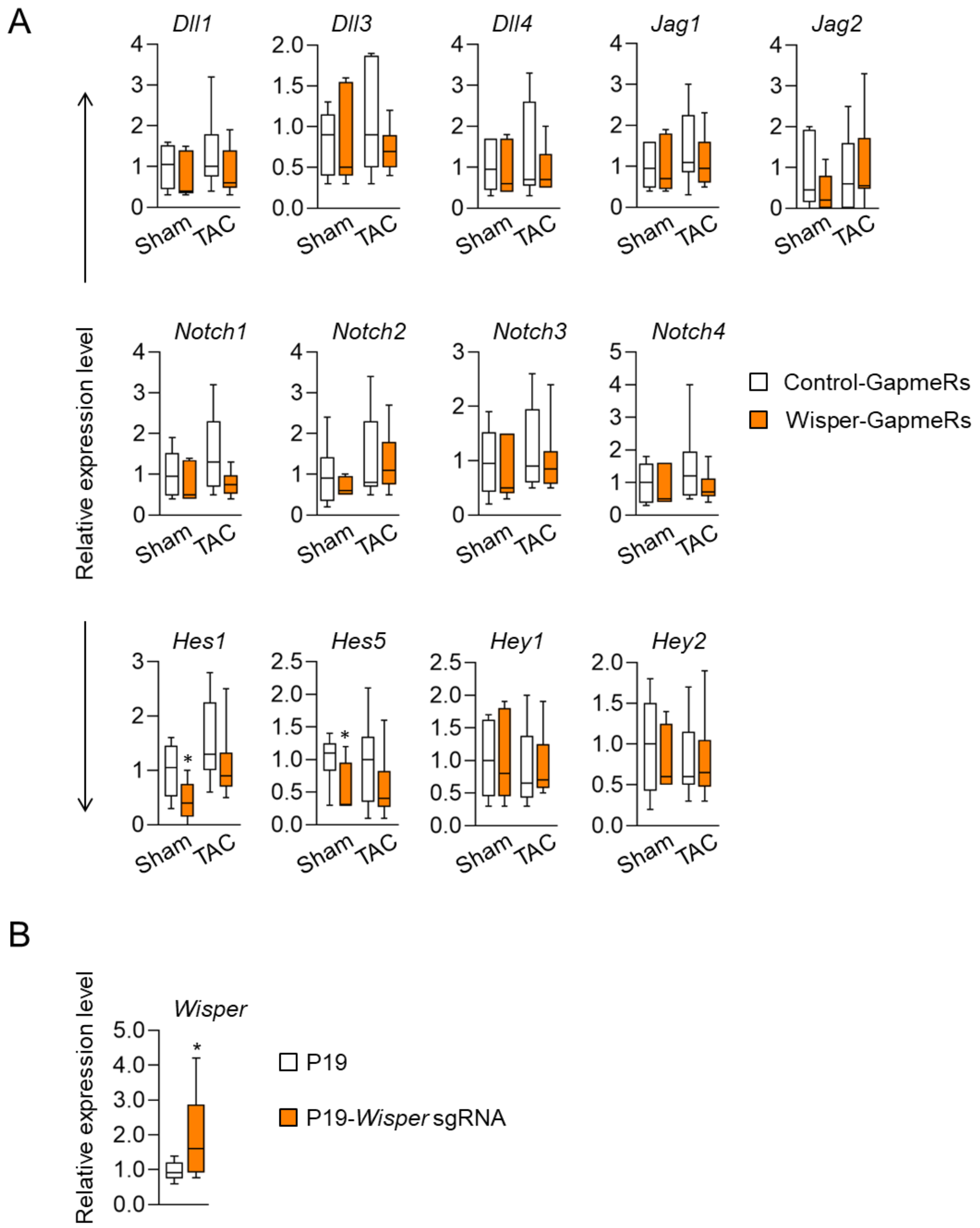

Supplementary Figure S5. Wisper knockdown is associated with downregulation of the NOTCH pathway in the heart. Mice were subjected to the experimental protocol as in Figure 5A. (**A**). Quantitative RT-PCR analysis of the expression level of NOTCH ligands (Dll1, Dll3, Dll4, Jag1 and Jag2), receptors (Notch1-Notch4) and target genes (Hes1, Hes5, Hey1 and Hey2). *, p<0.05 in Wisper-Gapmer vs. Control-GapmeR. (**B**). Activation of Wisper expression in P19Cl6 cell line. P19Cl6 embryonal carcinoma cells were transfected a plasmid encoding dCAs9-vp16-MS2-p65-Hsf1 and a plasmid expressing a guide RNA targeting Wisper promoter region to induce Wisper expression. The expression of Wisper was evaluated by quantitative RT-PCR analysis. Mean ± SEM *, p<0.05 in P19-Wisper sgRNA vs. P19, (n = 3).
